# Long-term ambient benzene exposure increases incident NAFLD risk through accelerated biological aging

**DOI:** 10.1016/j.isci.2026.117035

**Published:** 2026-09-30

**Authors:** Yaxiong Li, Ziwei Peng, Jing Zhang, Hong Cao, Jingyuan Zhang

**Affiliations:** 1 Department of Spine, Renmin Hospital, Hubei University of Medicine, Shiyan, Hubei, China; 2 Department of Neurosurgery, Renmin Hospital, Hubei University of Medicine, Shiyan, Hubei, China; 3 The Second Department of Infectious Disease, Shanghai Fifth People’s Hospital, Fudan University, Shanghai, China; 4 Center of Community-Based Health Research, Fudan University, Shanghai, China; 5 Department of Traumatic Orthopedics, Renmin Hospital, Hubei University of Medicine, Shiyan, China; 6 Jiangsu Key Laboratory for Biomaterials and Devices, School of Biological Science and Medical Engineering, Southeast University, Nanjing, China

**Keywords:** Nonalcoholic fatty liver disease, Benzene exposure, Environmental pollution, Biological aging, Polygenic risk score, Gene-environment interaction, UK Biobank

## Abstract

The contribution of ambient air pollutants to non-alcoholic fatty liver disease (NAFLD) remains undefined. This study included 401,472 adults from the UK Biobank (UKB), with residential benzene estimated at 1-km resolution from the DEFRA model (2006–2020). After multivariable adjustment in the Cox models, each interquartile-range (0.151 μg/m^3^) increment in benzene was associated with a 26% higher risk of incident NAFLD (hazard ratio [HR] = 1.26; 95% confidence interval [CI], 1.21–1.30), following a nonlinear exposure-response relationship. Accelerated biological aging mediated 6.5% of this association, whereas homeostatic dysregulation and allostatic load contributed modestly. Benzene exposure and polygenic risk showed no multiplicative interaction, but their cumulative effect raised NAFLD risk among highly susceptible individuals. A machine-learning model integrating benzene and covariates predicted NAFLD with good discrimination (AUC = 0.753), and an external urinary-biomarker analysis in NHANES was directionally consistent. Thus, long-term low-level benzene exposure increases incident NAFLD risk, partly through accelerated biological aging.

## Highlights

• Long-term ambient benzene exposure increased incident NAFLD risk by 26%
• The exposure-response relationship showed a nonlinear dose-dependent pattern
• Accelerated biological aging mediated 6.5% of the benzene-NAFLD association
• Genetic risk and benzene exposure exerted cumulative, not multiplicative, effects

## Graphical abstract

**Figure:**
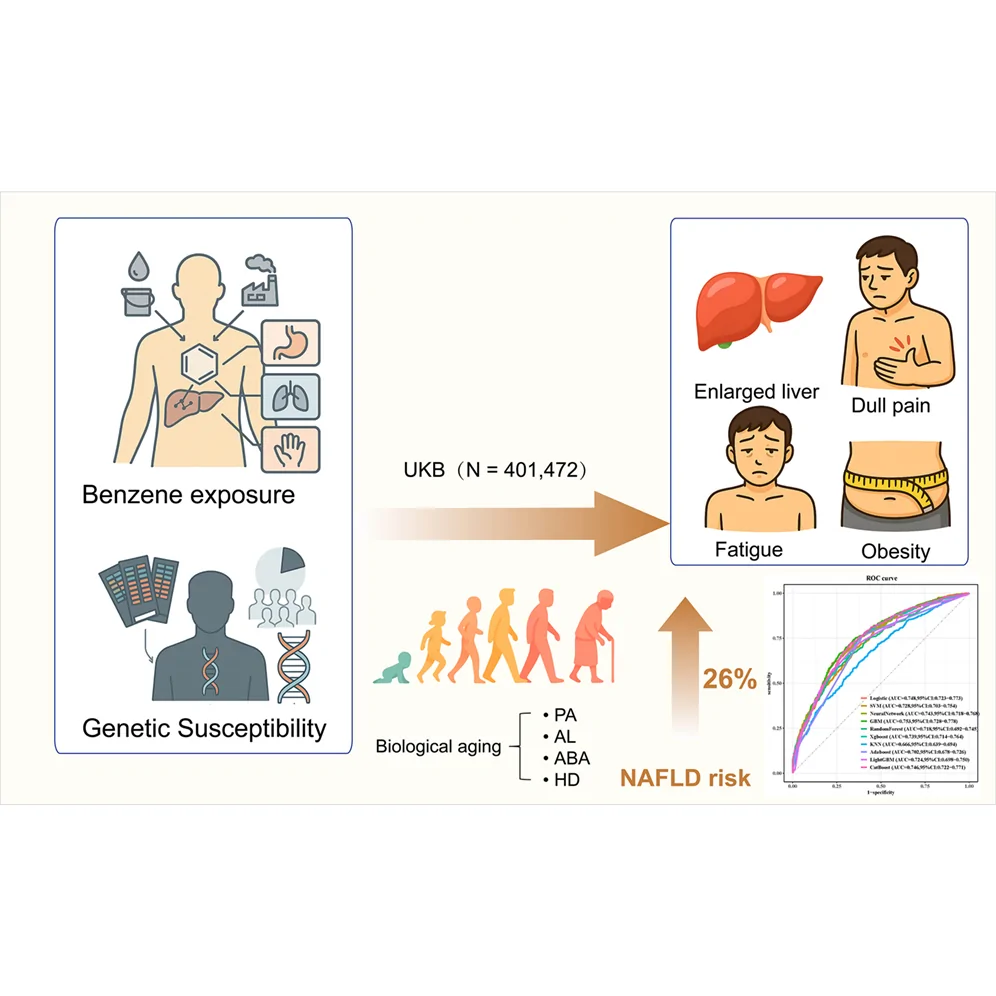

## Teaser

Health sciences; Environment

## Introduction

Non-alcoholic fatty liver disease (NAFLD) has become one of the most prevalent chronic liver disorders globally and represents a major contributor to the rising burden of liver-related morbidity in the coming decades. Teng et al.^1^ reported that the global annual incidence of NAFLD is approximately 47 cases per 1,000 individuals, with an overall adult prevalence of 32%, higher among men (40%) than women (26%). The prevalence has increased from 26% in 2005 to 38%, exceeding 40% in the Americas and Southeast Asia. Although obesity, insulin resistance, and metabolic syndrome are well-established risk factors, increasing attention has been directed toward the role of environmental exposures. Benzene, a widely distributed volatile organic compound (VOC) emitted from vehicular exhaust and industrial processes, can enter the human body chronically via inhalation and disrupt metabolic homeostasis. Indeed, a national English cohort recently linked long-term exposure to low concentrations of ambient benzene with elevated all-cause and cause-specific mortality.^2^ Recent multi-omics studies have demonstrated that benzene exposure induces mitochondrial dysfunction and reprograms lipid metabolism, leading to impaired oxidative phosphorylation, defective fatty acid oxidation, and insulin resistance.^3^^,^^4^ These metabolic disturbances closely mirror the core pathological mechanisms of NAFLD, suggesting that benzene may contribute to hepatic lipid accumulation by disturbing energy homeostasis and promoting chronic inflammation. However, prospective population-based studies examining the association between low-level environmental benzene exposure and NAFLD risk remain limited. The long-term health implications and underlying mechanisms of such exposure warrant further elucidation through large-scale epidemiological and mechanistic investigations.

Furthermore, individual genetic susceptibility may substantially influence the effects of environmental exposures on the risk of NAFLD. Recent large-scale genome-wide association study (GWAS) meta-analyses have identified 17 susceptibility loci—including PNPLA3, TM6SF2, GCKR, MBOAT7, APOE, and SREBF1—across multi-ethnic populations.^5^ These genes collectively regulate mitochondrial function, cholesterol biosynthesis, and lipid metabolism, thereby contributing causally to the genetic predisposition to NAFLD.^5^ Based on these findings, polygenic risk scores (PRSs) have been developed to quantify individual genetic risk and have shown strong predictive value for NAFLD susceptibility and related complications. De Vincentis et al.^6^ reported that integrating PRS with clinical fibrosis scores markedly improved the area under the receiver operating characteristic curve (AUC) for predicting severe liver diseases—including cirrhosis and hepatocellular carcinoma—by approximately 0.03–0.07 (*p* < 10^−3^). Similarly, Bianco et al.^7^ found that individuals with high PRS values exhibited nearly a 6-fold higher risk of developing hepatocellular carcinoma, indicating that genetic burden plays a pivotal role in the progression of metabolic liver diseases. However, despite robust evidence for the genetic contribution to NAFLD, its interaction with environmental exposures remains poorly characterized.

Benzene, a ubiquitous VOC in ambient air, has been shown to accelerate biological aging through oxidative stress and epigenetic reprogramming.^8^ Accelerated aging, in turn, is independently associated with an increased risk of NAFLD.^9^ Specifically, the continuous generation of reactive oxygen species during benzene metabolism may induce chronic oxidative stress, leading to the accumulation of DNA damage and progressive telomere shortening. In addition, benzene exposure can disrupt genome-wide DNA methylation patterns and alter histone modifications, thereby contributing to the acceleration of epigenetic age.^10^ These molecular injuries may further activate the senescence-associated secretory phenotype (SASP), promoting the sustained release of pro-inflammatory factors and creating a chronic low-grade inflammatory microenvironment.^11^^,^^12^ In the liver, accelerated hepatocyte aging is often accompanied by impaired mitochondrial function, reduced fatty acid oxidation, and compensatory upregulation of lipogenic pathways, ultimately facilitating hepatic lipid accumulation. Recent evidence has confirmed that accelerated biological aging is independently associated with an elevated risk of NAFLD,^13^ providing a theoretical basis for the hypothesis that benzene exposure may promote hepatic lipid deposition through accelerated biological aging. However, this mediation hypothesis has not yet been systematically validated in a large-scale prospective population.

Therefore, this study utilized long-term follow-up data from the UK Biobank and annual benzene concentrations estimated at a 1 km resolution using the DEFRA atmospheric dispersion and chemical transport model. By integrating PRSs and four biological aging-related indicators, we systematically investigated the association between long-term benzene exposure and the risk of developing NAFLD. Furthermore, we examined the potential interaction between genetic susceptibility and benzene exposure, as well as the mediating role of biological aging in this relationship.

## Results

### Baseline characteristics of the study population

Over a median follow-up of 12.4 years, 5,848 of the 401,472 UK Biobank participants developed incident NAFLD, Figure 1. Table 1 summarizes the baseline characteristics of the study population. The median age of participants was 58.0 years (interquartile range [IQR]: 50.0–63.0), and 54.5% were female. Overall, 94.8% of the participants were of White ethnicity. Compared with those without NAFLD, participants with NAFLD had a markedly higher prevalence of obesity (53.2% vs. 23.6%), hypertension (67.5% vs. 31.2%), diabetes (37.6% vs. 8.4%), and asthma (21.1% vs. 9.6%). In addition, participants with NAFLD were less likely to engage in regular physical activity (54.6% vs. 62.0%) and had a lower median healthy diet score (3.0 [IQR: 2.0–3.5]), Figure 2.

**Figure 1:**
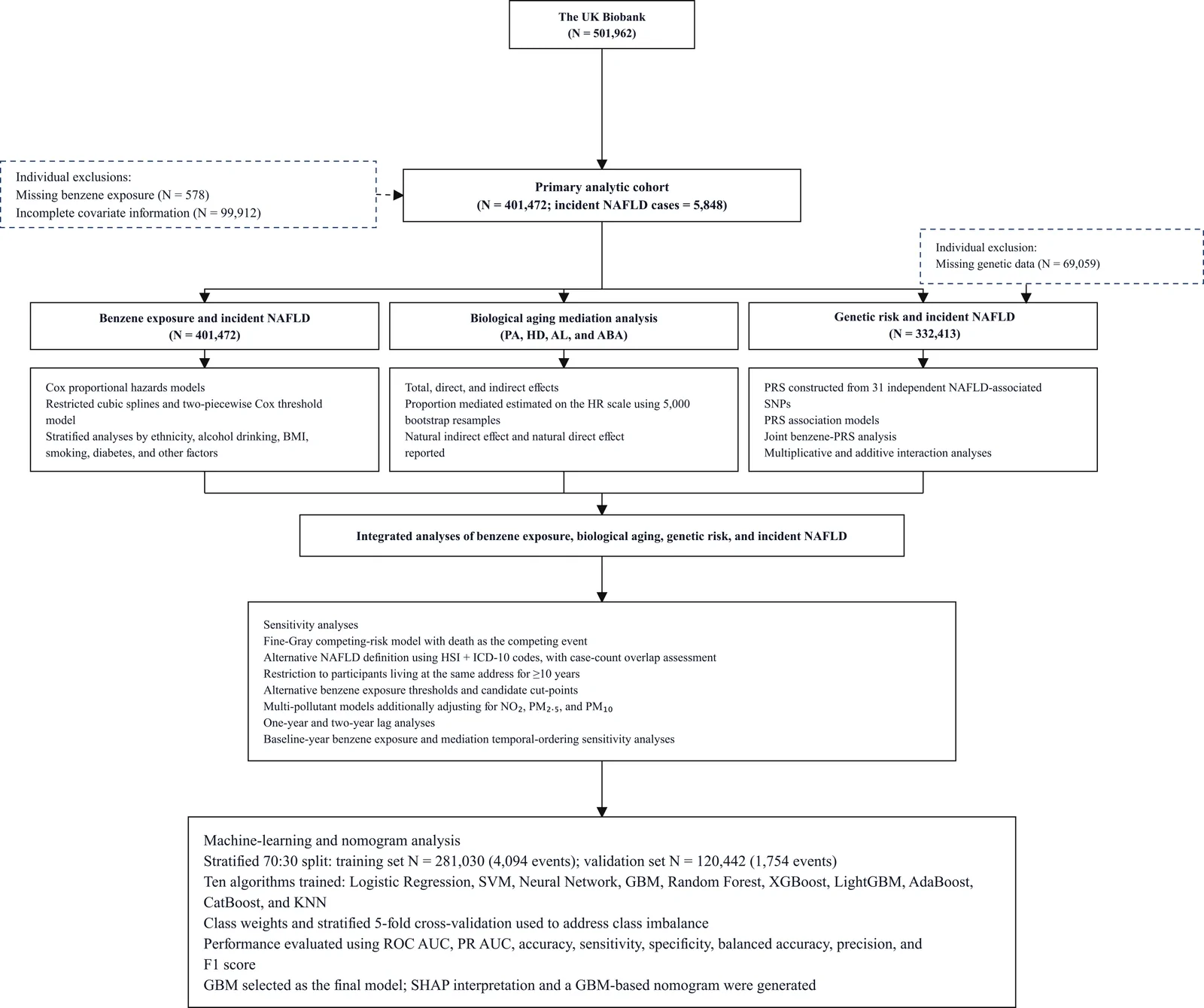
Study design and participant flow of the UK Biobank cohort This schematic summarizes participant inclusion, exposure assessment, genetic and biological-aging analyses, and the statistical and machine-learning procedures. A total of 401,472 participants were included for benzene-exposure analyses, and 332,413 with complete genetic data for PRS-related models.

**Table 1:** Baseline characteristics of 401,472 participants in the UK biobank study

Characteristic	Total population (*N* = 401,472)	NAFLD cases	
		no (*N* = 395,624)	yes (*N* = 5,848)
**Age**			

Median (Q1, Q3)	58.00 (50.00, 63.00)	58.00 (50.00, 63.00)	58.00 (51.00, 64.00)

**Gender**			

Female	218,893.00 (54.52%)	215,889.00 (54.57%)	3,004.00 (51.37%)
Male	182,579.00 (45.48%)	179,735.00 (45.43%)	2,844.00 (48.63%)

**Ethnicity**			

White	380,704.00 (94.83%)	375,206.00 (94.84%)	5,498.00 (94.02%)
Non White	20,768.00 (5.17%)	20,418.00 (5.16%)	350.00 (5.98%)

**BMI**			

Overweight	170,971.00 (42.59%)	168,843.00 (42.68%)	2,128.00 (36.39%)
Normal	131,859.00 (32.84%)	131,255.00 (33.18%)	604.00 (10.33%)
Obese	96,541.00 (24.05%)	93,433.00 (23.62%)	3,108.00 (53.15%)
Underweight	2,101.00 (0.52%)	2,093.00 (0.53%)	8.00 (0.14%)

**Education**			

levels	198,689.00 (49.49%)	195,639.00 (49.45%)	3,050.00 (52.15%)
College	132,272.00 (32.95%)	130,957.00 (33.10%)	1,315.00 (22.49%)
Unknown	70,511.00 (17.56%)	69,028.00 (17.45%)	1,483.00 (25.36%)

**Smoking status**			

Current	41,280.00 (10.28%)	40,434.00 (10.22%)	846.00 (14.47%)
Never	220,282.00 (54.87%)	217,652.00 (55.01%)	2,630.00 (44.97%)
Previous	139,910.00 (34.85%)	137,538.00 (34.76%)	2,372.00 (40.56%)

**Drinking status**			

Current	370,898.00 (92.38%)	365,781.00 (92.46%)	5,117.00 (87.50%)
Never	16,566.00 (4.13%)	16,218.00 (4.10%)	348.00 (5.95%)
Previous	14,008.00 (3.49%)	13,625.00 (3.44%)	383.00 (6.55%)

**Physical activity**			

Yes	248,377.00 (61.87%)	245,187.00 (61.97%)	3,190.00 (54.55%)
Non	153,095.00 (38.13%)	150,437.00 (38.03%)	2,658.00 (45.45%)

**Score diet**			

Median (Q1, Q3)	3.00 (2.00, 4.00)	3.00 (2.00, 4.00)	3.00 (2.00, 3.50)

**Household income**			

31,000 to 51,999	104,592.00 (26.05%)	103,089.00 (26.06%)	1,503.00 (25.70%)
Less than 18,000	91,830.00 (22.87%)	90,497.00 (22.87%)	1,333.00 (22.79%)
18,000 to 30,999	102,129.00 (25.44%)	100,648.00 (25.44%)	1,481.00 (25.32%)
52,000 to 100,000	81,329.00 (20.26%)	80,101.00 (20.25%)	1,228.00 (21.00%)
Greater than 100,000	21,592.00 (5.38%)	21,289.00 (5.38%)	303.00 (5.18%)

**Discoast**			

Median (Q1, Q3)	43.16 (13.33, 64.40)	43.16 (13.33, 64.40)	43.24 (13.12, 64.48)

**TDI**			

Median (Q1, Q3)	−2.17 (−3.66, 0.47)	−2.17 (−3.66, 0.47)	−2.14 (−3.66, 0.51)

**Hypertension**			

No	274,024.00 (68.25%)	272,121.00 (68.78%)	1,903.00 (32.54%)
Yes	127,448.00 (31.75%)	123,503.00 (31.22%)	3,945.00 (67.46%)

**Diabetes**			

No	365,881.00 (91.13%)	362,233.00 (91.56%)	3,648.00 (62.38%)
Yes	35,591.00 (8.87%)	33,391.00 (8.44%)	2,200.00 (37.62%)

**Asthma**			

No	362,342.00 (90.25%)	357,729.00 (90.42%)	4,613.00 (78.88%)
Yes	39,130.00 (9.75%)	37,895.00 (9.58%)	1,235.00 (21.12%)

**Sleep duration**			

7∼8	263,160.00 (65.55%)	259,864.00 (65.68%)	3,296.00 (56.36%)
<7	105,712.00 (26.33%)	103,821.00 (26.24%)	1,891.00 (32.34%)
>8	32,600.00 (8.12%)	31,939.00 (8.07%)	661.00 (11.30%)

**Benzene, μg/m**^**3**^			

Median (Q1, Q3)	0.43 (0.35, 0.50)	0.43 (0.35, 0.50)	0.44 (0.37, 0.52)
PhenoAge			
Median (Q1, Q3)	43.55 (36.27, 49.74)	43.52 (36.24, 49.71)	45.49 (38.25, 51.50)
HD			
Median (Q1, Q3)	4.98 (3.92, 5.93)	4.99 (3.93, 5.94)	4.45 (3.42, 5.38)
AL			
Median (Q1, Q3)	0.22 (0.11, 0.33)	0.22 (0.11, 0.33)	0.33 (0.22, 0.44)
ABA			
Median (Q1, Q3)	−0.29 (−3.61, 3.19)	−0.33 (−3.65, 3.14)	2.71 (−0.60, 6.26)

ABA, accelerated biological aging; AL, allostatic load; BMI, body mass index; Discoast, distance from residence to the coast; HD, homeostatic dysregulation; NAFLD, nonalcoholic fatty liver disease; TDI, Townsend Deprivation Index.

**Figure 2:**
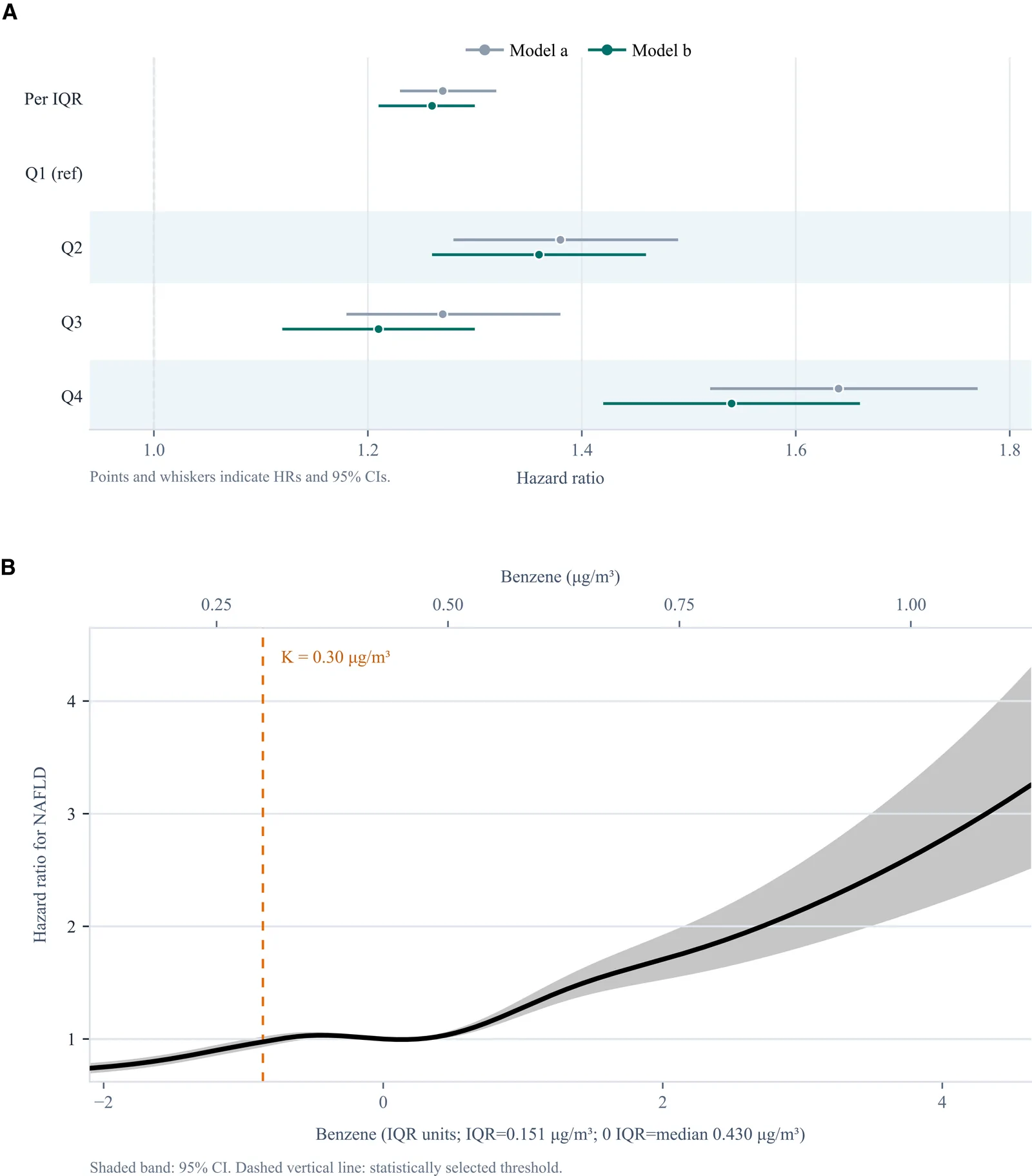
Association between long-term residential benzene exposure and incident NAFLD in the UK Biobank (*n* = 401,472) (A) Hazard ratios (HRs) per interquartile-range (IQR = 0.151 μg/m3) increment and across exposure quartiles (Q1, reference). (B) Restricted cubic spline curve with knots at the 10^th^, 50^th^, and 90^th^ percentiles; the orange dashed line marks the inflection point (K = 0.30 μg/m3) from a two-piecewise Cox model. Model a was adjusted for age and sex; model b was further adjusted for ethnicity, body mass index, education level, household income, smoking status, alcohol drinking status, physical activity, healthy diet score, residential distance to the coast, Townsend Deprivation Index, hypertension, diabetes, asthma, and sleep duration. Points and whiskers indicate HRs and 95% CIs; the shaded band in (B) indicates the 95% CI. Estimates were derived from multivariable Cox proportional hazards models (*n* = 401,472; 5,848 incident NAFLD events). CI, confidence interval; HR, hazard ratio; IQR, interquartile range; Q, quartile.

### Association between long-term residential benzene exposure and incident NAFLD

In the multivariable-adjusted Cox proportional hazards model (Table 2), higher benzene exposure was significantly associated with an increased risk of NAFLD. For each IQR increment of benzene concentration (0.151 μg/m^3^), the risk of NAFLD increased by 26% (adjusted hazard ratio [HR] = 1.26; 95% confidence interval [CI], 1.21–1.30; *p* = 2.27 × 10^−40^). When participants were categorized by exposure quartiles, compared with the lowest exposure group (Q1), the risk of NAFLD was 36% higher in Q2 (HR = 1.36; 95% CI, 1.26–1.46), 21% higher in Q3 (HR = 1.21; 95% CI, 1.12–1.30), and 54% higher in Q4 (HR = 1.54; 95% CI, 1.42–1.66) (*p* for trend<0.001). Threshold analysis (Table 3; Figure 3) demonstrated a nonlinear dose-response association between benzene exposure and NAFLD risk, with an inflection point around 0.3 μg/m^3^. Below this threshold, benzene exposure showed a modest positive association with NAFLD risk (HR = 1.08; 95% CI, 1.07–1.09). Above 0.3 μg/m^3^, the association became markedly stronger (HR = 1.29; 95% CI, 1.22–1.37; *p* < 0.001).

**Table 2:** Adjusted hazard ratios for NAFLD by benzene exposure

Pollution^a^^,^^b^^,^^c^	Increase	Events	Total/person-years	HR (95% CI)		
				Model a	Model b	*p* for trend
Benzene, μg/m^3^	per IQR increment	5,848	401,472/5,322,586.649	1.27 (1.23–1.32)	1.26 (1.21–1.30)	2.27 × 10^−40^
–	Q1 (ref)	1,164	99,206/1,363,073.123	Ref.	Ref.	–
–	Q2 (0.353,0.43]	1,577	98,789/1,347,240.816	1.38 (1.28,1.49)	1.36 (1.26,1.46)	–
–	Q3 (0.43,0.504]	1,438	98,938/1,336,796.630	1.27 (1.18,1.38)	1.21 (1.12,1.30)	–
–	Q4 (0.504,1.13]	1,669	98,691/1,275,476.079	1.64 (1.52,1.77)	1.54 (1.42,1.66)	–

a Model a: Cox proportional hazards model adjusted for age and sex.

b Model b: Cox proportional hazards model adjusted for age, sex, ethnicity, BMI, education level, smoking status, alcohol drinking status, physical activity, household income, healthy diet score, distance from residence to the coast, TDI, hypertension, diabetes, asthma, and sleep duration.

c NAFLD, nonalcoholic fatty liver disease; BMI, body mass index; CI, confidence interval; HR, hazard ratio; Q, quartile; TDI, Townsend Deprivation Index; μg/m^3^, micrograms per cubic meter.

**Table 3:** Threshold associations of benzene with NAFLD

NAFLD	Participants (%)	Events	Person-years	HR (95% CI)	*p* Value
Continuous, per IQR	401,472 (100.0)	5,848	5,322,586.649	1.26 (1.21, 1.30)	2.27 × 10ˆ-40
Inflection point (K)	0.30 μg/m^3^	–	–	–	–
<K	98,961 (24.7)	1,169	1,312,264.820	1.08 (1.07–1.09)	<0.001
≥K	302,511 (75.3)	4,679	4,010,321.829	1.29 (1.22–1.37)	<0.001
Likelihood ratio test for threshold model	–	–	–	–	0.008

Hazard ratios (HRs) and 95% confidence intervals (CIs) were estimated using a two-piecewise Cox proportional hazards regression model. The inflection point (K) represents the threshold level of benzene exposure at which the relationship with NAFLD risk changes slope.

**Figure 3:**
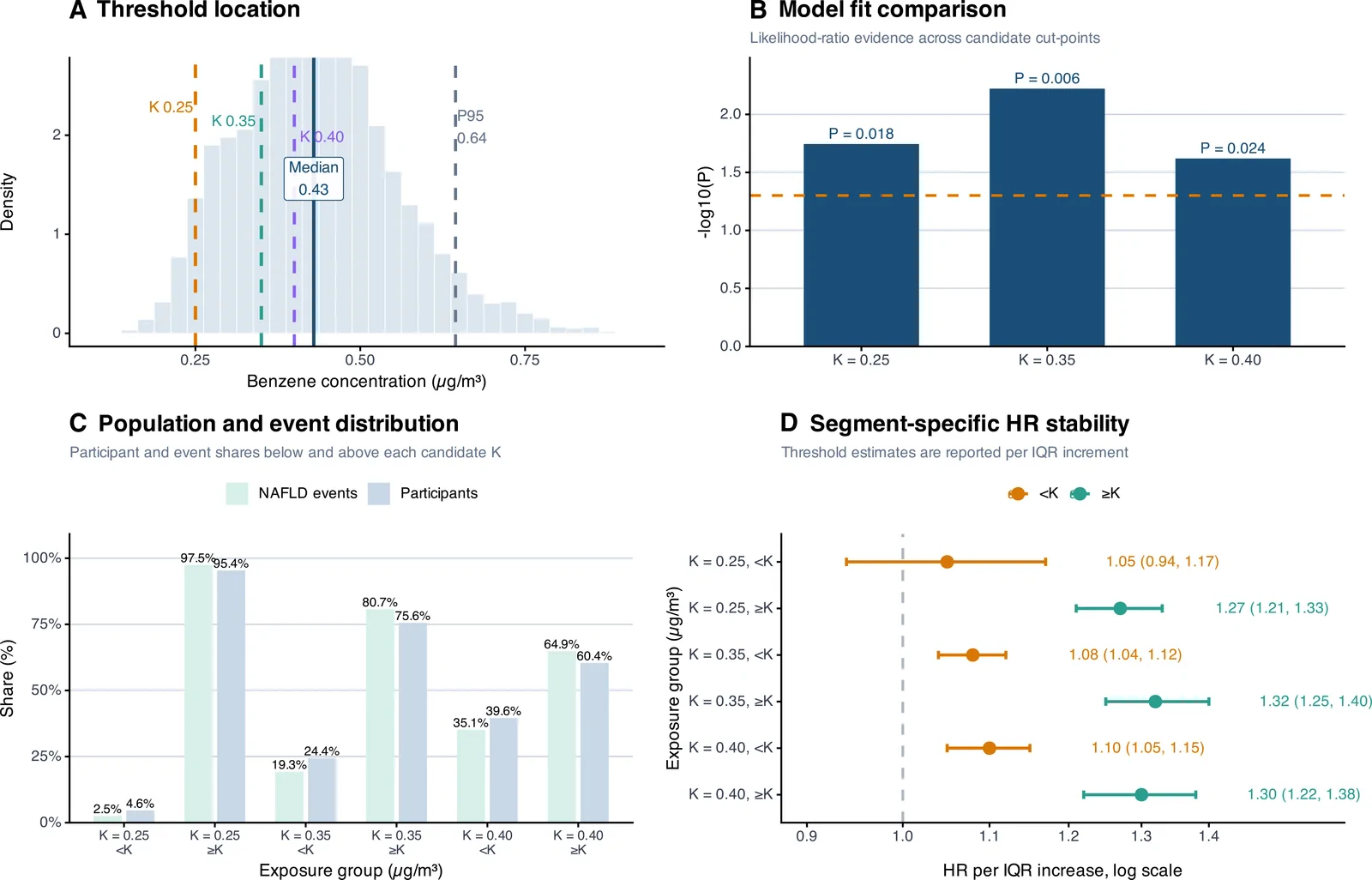
Robustness of the benzene-NAFLD inflection point across alternative candidate cut-points (K = 0.25, 0.35, and 0.40 μg/m3) in the UK Biobank (*n* = 401,472) (A) Density of long-term residential benzene concentration overlaid with candidate cut-points, the cohort median (0.43 μg/m3), and the 95^th^ percentile (0.64 μg/m3). (B) −log10 *p* values from likelihood-ratio tests against the single-line Cox model; the dashed line denotes *p* = 0.05. (C) Shares of participants and incident NAFLD events below (<K) and above (≥ K) each cut-point. (D) Segment-specific HRs (95% CIs) per IQR (0.151 μg/m3) increment, on a log scale. HRs were estimated using two-piecewise Cox proportional hazards models.

### Subgroup and stratified analyses

In the stratified analysis (Table 4), the association between benzene exposure and NAFLD risk was generally consistent across subgroups, with no evidence of significant heterogeneity in most strata (all *p* for heterogeneity >0.05), suggesting strong robustness of the results. The association appeared slightly stronger among non-White participants than among White participants (HR = 1.33; 95% CI, 1.10–1.61 vs. HR = 1.23; 95% CI, 1.19–1.27). When stratified by alcohol consumption, current drinkers exhibited the highest risk (HR = 1.31; 95% CI, 1.29–1.33; *p* for heterogeneity = 0.044). The risk was also modestly higher among individuals with diabetes (HR = 1.23; 95% CI, 1.18–1.28; *p* for heterogeneity = 0.027). Across other subgroups, including body mass index (BMI), smoking status, physical activity, asthma, and sleep duration, the direction of association remained consistent, and no significant heterogeneity was observed.

**Table 4:** Subgroup analyses of benzene and NAFLD

Subgroup	Level	Events	Total/Person years	HR (95% CI)	*p* Heterogeneity
Ethnicity	White	5498	380,704/5,051,455	1.23 (1.19–1.27)	0.438
	non-White	350	20,768/271,131	1.33 (1.10–1.61)	
Alcohol drinking	current	5117	370,898/4,925,757	1.31 (1.29–1.33)	0.044
	never	348	16,566/217,561	1.21 (1.17–1.26)	
	previous	383	14,008/179,268	1.30 (1.15–1.45)	
BMI	overweight	2128	170,971/2,271,235	1.23 (1.17–1.30)	0.095
	normal	604	131,859/1,762,438	1.33 (1.26–1.40)	
	obese	3108	96,541/1,261,929	1.22 (1.16–1.27)	
	underweight	8	2,101/26,983	1.11 (0.32–3.86)	
Smoking	current	846	41,280/533,071	1.28 (1.18–1.39)	0.199
	never	2630	220,282/2,951,102	1.26 (1.20–1.32)	
	previous	2372	139,910/1,838,414	1.19 (1.13–1.25)	
Regular physical activity	yes	3190	248,377/3,301,997	1.23 (1.18–1.29)	0.827
	no	2658	153,095/2,020,590	1.24 (1.18–1.30)	
Diabetes	no	3648	365,881/4,873,163	1.14 (1.08–1.21)	0.027
	yes	2200	35,591/449,424	1.23 (1.18–1.28)	
Hypertension	no	1903	274,024/3,668,221	1.23 (1.16–1.30)	0.526
	yes	3945	127,448/1,654,365	1.17 (1.12–1.22)	
Asthma	no	4613	362,342/4,810,557	1.22 (1.17–1.26)	0.333
	yes	1235	39,130/512,030	1.26 (1.17–1.36)	
Sleep duration	7–8	3296	263,160/3,500,425	1.20 (1.15–1.26)	0.067
	<7	1891	105,712/1,398,225	1.31 (1.23–1.38)	
	>8	661	32,600/423,936	1.18 (1.07–1.30)	

### PRS and joint effect of benzene exposure and genetic susceptibility

The results of the PRS analysis are presented in Table 5. Each one-standard deviation increase in PRS was associated with a 36% higher risk of NAFLD (HR = 1.36; 95% CI, 1.30–1.43; *p* = 1.07 × 10–33). Compared with participants in the lowest genetic risk tertile, those in the intermediate and high tertiles had 13% (HR = 1.13; 95% CI, 1.06–1.22) and 44% (HR = 1.44; 95% CI, 1.34–1.54) higher risks of NAFLD, respectively. In the joint analysis of benzene exposure and genetic susceptibility (Table S5), participants with both high genetic risk and high benzene exposure had the highest risk of NAFLD (HR = 1.73; 95% CI, 1.56–1.91) compared with those with low genetic risk and low exposure. The additive interaction analysis (Table S8) showed a significant positive interaction (relative excess risk due to interaction [RERI] = 0.27, 95% CI 0.06–0.48; AP = 0.16, 95% CI 0.04–0.28; synergy index [SI] = 1.46, 95% CI 1.08–1.98), indicating that benzene exposure and genetic risk act synergistically. The distribution of benzene exposure and key covariates was balanced across PRS tertiles (Table S6), and all PRS-related associations remained consistent when analyses were restricted to the genetically defined European-ancestry subgroup (Tables S9–S12).

**Table 5:** The association between polygenic risk score and incident NAFLD

Genetic risk category	Events	Total/Person years	Model a		Model b		Model c	
			HR (95% CI)	*p* Value	HR (95% CI)	*p* Value	HR (95% CI)	*p* Value
Polygenic risk score	5,104	332,413/4,378,543.551	1.36 (1.30–1.43)	1.07 × 10^−33^	1.34 (1.29–1.42)	3.92 × 10^−32^	1.35 (1.29–1.42)	4.42 × 10^−32^
Low Genetic Risk	1,432	110,879/1,462,466.142	Ref.	–	Ref.	–	Ref.	–
Intermediate Genetic Risk	1,622	110,764/1,459,902.370	1.13 (1.06–1.22)	5.22 × 10^−4^	1.10 (1.03–1.19)	0.005	1.11 (1.03–1.19)	0.005
High Genetic Risk	2,050	110,770/1,456,175.038	1.44 (1.34–1.54)	5.63 × 10^−26^	1.41 (1.32–1.52)	4.80 × 10^−24^	1.42 (1.32–1.52)	4.70 × 10^−24^

Model a was adjusted for age, sex, and the first 10 genetic principal components. Model b was adjusted for age, sex, ethnicity, BMI, education level, household income, smoking status, alcohol drinking status, healthy diet score, physical activity, distance from residence to the coast, TDI, hypertension, diabetes, asthma, sleep duration, and the first 10 genetic principal components. Model c was further adjusted for benzene exposure. Abbreviations: BMI, body mass index; CI, confidence interval; HR, hazard ratio; NAFLD, nonalcoholic fatty liver disease; PC, principal component; PRS, polygenic risk score; TDI, Townsend Deprivation Index.

### Mediation by accelerated biological aging

The mediation analysis included participants with available measurement dates for biological aging indicators. The results showed that the total effect of benzene exposure on NAFLD risk was statistically significant across all four biological aging indicator models (all *p* < 0.001) (Table S13). Specifically, ABA showed the most pronounced mediating effect, with a mediation proportion of 6.53% (95% CI: 4.73%–9.56%); the corresponding mediation proportions for HD and AL were 3.14% (95% CI: 1.83%–5.58%) and 2.52% (95% CI: 1.72%–3.80%), respectively. Further effect decomposition showed that the natural indirect effects of ABA, HD, and AL were 1.0109 (95% CI: 1.0079–1.0160), 1.0052 (95% CI: 1.0030–1.0093), and 1.0042 (95% CI: 1.0029–1.0063), respectively, suggesting that these biological aging indicators may partially mediate the association between benzene exposure and NAFLD risk. In contrast, PhenoAge (PA) showed a mediation proportion of −0.91% (95% CI: −2.33%–0.21%) and a natural indirect effect of 0.9985 (95% CI: 0.9959–1.0003), providing no stable statistical evidence of mediation.

### Sensitivity analyses

Sensitivity analyses supported the robustness of the main findings (Tables S14–S18 and S20–S23). The association remained significant after accounting for death as a competing event (subdistribution hazard ratio (SHR) = 1.24, 95% CI: 1.20–1.28; Table S14), redefining NAFLD using the combined HSI and Classification of Diseases, Tenth Revision (ICD-10) definition (HR = 1.36, 95% CI: 1.35–1.37; Tables S15 and S16), and restricting the analysis to participants living at the same address for at least 10 years (HR = 1.28, 95% CI: 1.22–1.33; Table S17). Results were also consistent after excluding NAFLD cases occurring within the first 1 or 2 years of follow-up (HR = 1.25 and 1.24, respectively; Table S22). Additional analyses using alternative benzene thresholds showed similar results (Tables S18 and S21). In multipollutant models, adjustment for NO2, PM2.5, and PM10 attenuated the association, but benzene exposure remained significantly associated with NAFLD risk even in the fully adjusted four-pollutant model (HR = 1.13, 95% CI: 1.06–1.20; Table S20). Finally, sensitivity analyses addressing the temporal ordering of mediation showed consistent indirect effects for HD, AL, and ABA, whereas PA showed no stable mediating effect (Table S23).

### Machine-learning prediction of incident NAFLD

Among the 10 machine learning algorithms tested, the gradient boosting machine (GBM) model demonstrated the best predictive performance, with an ROC AUC of 0.753 (95% CI: 0.728–0.778) and a PR AUC of 0.682 in the validation set, outperforming logistic regression (ROC AUC = 0.748) and CatBoost (ROC AUC = 0.746) (Table S25; Figure 4). For threshold-specific classification metrics, the GBM model yielded an accuracy of 0.705, sensitivity of 0.662, specificity of 0.736, and balanced accuracy of 0.699 (Table S26). Shapley additive explanation (SHAP) analysis showed that PhenoAge acceleration residual ranked as the most important predictive feature, followed by gender, BMI, physical activity, diabetes, HD, PhenoAge, benzene exposure, and age (Figure 4B). A nomogram constructed based on these variables was used for individualized risk estimation (Figure 4C), with an AUC of 0.705 in the validation set (Figure 4D), indicating that the nomogram had a certain discriminatory ability. In an external-consistency analysis using NHANES (*n* = 1,681), higher urinary trans-muconic acid (TTMA) was associated with greater odds of HSI-defined hepatic steatosis (odds ratio [OR] = 1.14; 95% CI, 1.04–1.24), directionally consistent with the UK Biobank findings (Tables S27, S28, and S29).

**Figure 4:**
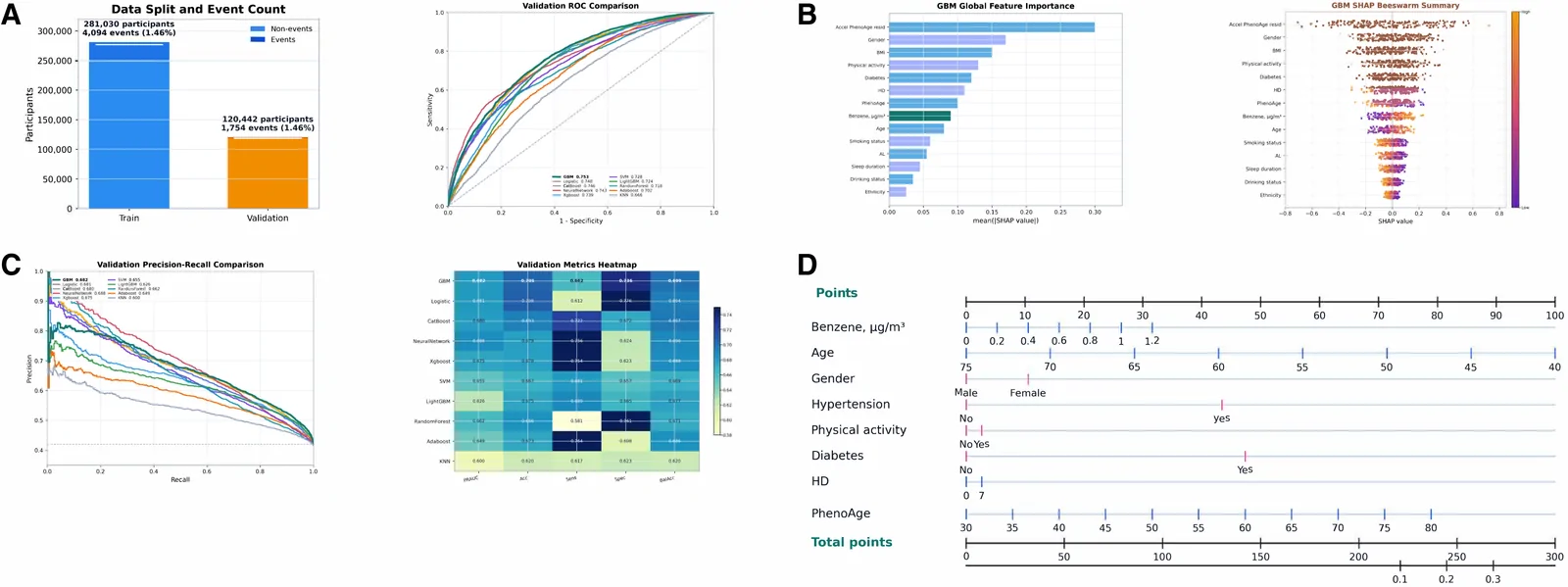
Machine-learning prediction of incident NAFLD using benzene exposure with biological-aging and clinical features (A) Training-validation data split (left) and validation ROC curves for ten algorithms (right). (B) Global feature importance by mean absolute SHAP value (left) and SHAP beeswarm summary (right) for the gradient boosting machine (GBM) model. (C) Validation precision-recall curves (left) and a heatmap of validation metrics (right). (D) GBM-derived nomogram for individualized NAFLD risk prediction. Models were trained on 70% of the analytic cohort (*n* = 281,030; 4,094 events) and evaluated on the held-out validation set (*n* = 120,442; 1,754 events); hyperparameters were tuned by stratified 5-fold cross-validation, with class weights inversely proportional to class frequency. AUC, area under the curve; GBM, gradient boosting machine; ROC, receiver operating characteristic; SHAP, Shapley additive explanations.

## Discussion

Among the 401,472 participants in the UK Biobank, we observed a significant positive association between long-term environmental benzene exposure and the risk of NAFLD, with evidence suggesting a potential non-linear exposure-response pattern. In addition, genetic susceptibility was independently associated with NAFLD risk, and a significant positive additive interaction was detected between benzene exposure and genetic risk. Mediation analysis further demonstrated that the effect of chronic benzene exposure on NAFLD was partially mediated by accelerated biological aging, with consistent directional effects observed for homeostatic dysregulation and allostatic load. These findings suggest that systemic aging may represent a key biological pathway through which environmental chemical exposure contributes to hepatic metabolic injury. Multiple sensitivity analyses—including models accounting for competing risks, alternative NAFLD definitions, restriction to long-term residents, and varying exposure thresholds—yielded consistent results, indicating that the direction and magnitude of the observed associations remained robust.

Apart from the study by Wang et al., prospective evidence on the relationship between air pollution and NAFLD remains scarce. In a cohort of 313,102 participants from the UK Biobank, Wang and colleagues reported significant associations between exposure to PM_2.5_, NO_2_, and NO_x_ and the risk of NAFLD, with HRs of 1.33, 1.38, and 1.33 for the highest exposure groups, respectively. BMI partially mediated these associations, with mediation proportions ranging from 2.9% to 18.8%. However, that study did not account for volatile organic compounds such as benzene.^14^^,^^15^ In contrast, the present study, involving 401,472 UK Biobank participants, is the first to demonstrate a significant association between long-term benzene exposure and NAFLD risk (HR = 1.26; 95% CI, 1.21–1.30). Moreover, accelerated biological aging was found to mediate 6.5% of this association. Compared with studies identifying BMI as a mediator, our findings highlight systemic aging as a novel biological pathway through which environmental exposure contributes to metabolic liver disease. An external analysis in NHANES, using urinary TTMA as a benzene biomarker, showed a directionally consistent association with hepatic steatosis; however, because the exposure metric and outcome definition differed and the association attenuated after adjustment for cotinine and creatinine, this finding should be regarded as supportive external-consistency evidence rather than definitive replication.

The mediating role of biological aging in the association between long-term benzene exposure and NAFLD is biologically plausible. Benzene and its metabolites can accelerate systemic aging processes and disrupt hepatic metabolic homeostasis by inducing oxidative stress, mitochondrial dysfunction, and chronic inflammatory responses. Epidemiological evidence has demonstrated a dose-dependent relationship between benzene exposure and epigenetic age acceleration; in occupationally exposed populations, individuals with high benzene exposure exhibit an average epigenetic age advancement of approximately 2.1 years.^10^ This accelerated aging state is closely linked to impaired energy metabolism, immune activation, and reduced cellular repair capacity-core pathophysiological features of NAFLD. The biological aging indicator, PhenoAge, integrates multiple clinical biomarkers across physiological systems and effectively captures systemic inflammation, oxidative stress, and metabolic load. Liu et al.^16^ validated PhenoAge in the NHANES cohort, demonstrating its robust predictive ability for all-cause mortality and metabolic diseases across diverse populations. Recent findings further indicate that each one-year increase in PhenoAge is associated with an 8% higher risk of liver fibrosis,^17^ suggesting that accelerated aging may represent an essential biological pathway underlying hepatic metabolic injury. In addition, HD and AL, as quantitative markers of systemic aging, reflect multisystem physiological deviation and cumulative chronic stress.^18^ In the present study, accelerated biological aging mediated 6.5% of the association between benzene exposure and NAFLD, with HD and AL showing consistent directional effects.^19^ These results suggest that benzene exposure may promote hepatic lipid accumulation by perturbing homeostatic networks, enhancing oxidative and inflammatory burdens, and accelerating aging-related metabolic decline. Experimental evidence supports this pathway: in C57BL/6J mice, Zhang et al. reported that 12 weeks of low-dose benzene inhalation (1 mg/kg) markedly increased hepatic IL-6 and TNF-α levels (2.3-fold and 2.8-fold, respectively; *p* < 0.01), accompanied by a 35% reduction in mitochondrial membrane potential and a 1.9-fold rise in malondialdehyde (MDA) concentration.^19^^,^^20^^,^^21^ These findings collectively indicate that benzene exposure induces oxidative stress-mediated mitochondrial injury and inflammatory activation, thereby accelerating hepatocellular metabolic aging. Beyond the liver, environmental stressors and genetic predisposition have been jointly linked to aging-related outcomes in other organ systems, such as auditory aging, underscoring the broad relevance of environment-aging interactions across tissues.^22^^,^^23^

The stratified analysis in this study demonstrated that the association between benzene exposure and the risk of NAFLD remained generally consistent across population subgroups, indicating the robustness of the association. Furthermore, a PRS for NAFLD was constructed to examine its joint effect and interaction with benzene exposure. Participants with both high genetic risk and high benzene exposure exhibited the highest incidence of NAFLD (HR = 1.73; 95% CI, 1.56–1.91), and the benzene-genetic association departed significantly from additivity (RERI = 0.27, 95% CI 0.06–0.48), although no significant multiplicative interaction was observed, indicating a synergistic gene-environment effect on the additive scale.^24^ A comparable pattern has been reported for ambient air pollutants, whose associations with disease risk were strengthened by genetic susceptibility.^25^ Mechanistically, genetic and environmental factors may act synergistically through partially overlapping metabolic and inflammatory pathways: PNPLA3 and TM6SF2 variants enhance oxidative stress and lipid-droplet accumulation, while benzene exposure similarly induces hepatic lipid metabolic disruption and inflammatory responses,^26^^,^^27^^,^^28^ so that benzene exposure may amplify the hepatic injury conferred by high genetic risk. Notably, currently identified GWAS loci explain only about 7.5% of the heritable variance in NAFLD and do not fully capture non-coding or epigenetic regulation; the PRS based on known loci may therefore underestimate genetic susceptibility, suggesting that the true gene-environment interaction could be even larger than observed.^29^

From a biological perspective, the association between benzene exposure and NAFLD is supported by a strong pathological foundation. Mechanistically, benzene and its metabolites—such as benzoquinone and hydroquinone—are metabolized in the liver by CYP2E1, generating ROS and weakening mitochondrial antioxidant defenses.^30^^,^^31^ These effects lead to lipid peroxidation, oxidative injury, and disturbances in hepatic energy metabolism. Benzene metabolites also activate the NF-κB and p38-MAPK signaling pathways, triggering inflammatory cascades that promote cytokine release and exacerbate lipid accumulation in hepatocytes.^32^ Correspondingly, elevated serum transaminase and alkaline phosphatase levels have been observed in benzene-exposed populations.^33^ Beyond oxidative injury, long-term benzene exposure is closely linked to epigenetic reprogramming and accelerated biological aging. Occupational studies have demonstrated that exposure to benzene and formaldehyde induces aberrant methylation at immune- and metabolism-related CpG sites,^34^ showing a dose-dependent acceleration of DNA methylation clocks.^10^ Similarly, telomere studies have revealed that chronic high-level benzene exposure is associated with leukocyte telomere shortening,^35^^,^^36^ implicating disrupted cellular replication and senescence pathways. Collectively, these oxidative and epigenetic alterations destabilize hepatic metabolic homeostasis. Notably, key metabolic regulators such as peroxisome proliferator-activated receptor alpha (PPARα) and sirtuin 1 (SIRT1) play pivotal roles in this process—the former governs fatty acid oxidation, whereas the latter preserves mitochondrial integrity and suppresses inflammatory transcriptional activity.^37^ Together, these findings support a cascade mechanism in which benzene exposure accelerates hepatic physiological aging and lipid deposition through an integrated oxidative stress-inflammation-epigenetic remodeling-biological aging-metabolic imbalance pathway. The mediating effects of ABA, HD, and AL observed in this study further substantiate systemic homeostasis disruption as a central mechanism linking benzene exposure to NAFLD development and progression.

Taken together, this large-scale prospective cohort study provides the first evidence linking long-term low-level benzene exposure to an increased risk of NAFLD, and demonstrates that this association is partially mediated by accelerated biological aging. Our findings underscore that environmental benzene exposure represents an important exogenous risk factor beyond traditional metabolic determinants and may contribute to hepatic metabolic decline by inducing systemic inflammation, disrupting homeostatic balance, and promoting phenotypic aging. These results highlight the potential role of environmental toxicants in the pathogenesis of metabolic liver diseases and emphasize the importance of incorporating aging-related pathways into environmental health risk assessment.

### Limitations of the study

This study has several limitations that should be acknowledged. First, although our sensitivity analyses further adjusted for major traffic-related co-pollutants, benzene exposure itself occurs as part of a complex mixture of VOCs; the contributions of unmeasured co-exposures—including toluene, xylene, and sulfur dioxide—cannot be fully disentangled, and residual confounding therefore remains possible. Second, incident NAFLD was ascertained primarily from inpatient ICD codes, an outcome definition that may miss non-hospitalized or early-stage cases; in addition, code K75.8 may capture some atypical liver diseases, introducing a degree of outcome misclassification. Third, our models adjusted only for baseline self-reported drinking status rather than for specific thresholds of daily alcohol intake, owing to the self-reported nature and intrinsic measurement error of the UK Biobank alcohol-frequency and alcohol-volume data. This may have led to the inclusion of some participants whose actual alcohol intake exceeded the recommended limits. Future studies should incorporate more refined alcohol-quantification tools or serum biomarkers to improve the diagnostic specificity of NAFLD. In addition, several lifestyle- and diet-related covariates were drawn from baseline self-administered questionnaires; these variables are susceptible not only to measurement error but also to change during follow-up, so residual confounding cannot be completely excluded despite extensive adjustment. Finally, UK Biobank participants are generally healthier than the general population, introducing a degree of healthy-volunteer bias.

## Resource availability

### Lead contact

Requests for further information and resources should be directed to and will be fulfilled by the lead contact, Jingyuan Zhang (zjy666223@163.com).

### Materials availability

This study did not generate new unique reagents.

### Data and code availability

• Individual-level UK Biobank data are available to approved researchers through the UK Biobank Access Management System (https://www.ukbiobank.ac.uk/; this study was conducted under application number 694569). Ambient benzene concentration data (1 km grid, 2006–2020) are publicly available from the DEFRA UK-AIR archive (https://uk-air.defra.gov.uk). NHANES 2017-March 2020 data are publicly available from the US. CDC/NCHS (https://www.cdc.gov/nchs/nhanes/). Accession details are also listed in the key resources table.
• All statistical analyses were performed using the FastUKB R package; this paper does not report original custom code. Requests for the underlying source code should be directed to the developers of the FastUKB package.
• Any additional information required to reanalyze the data reported in this paper is available from the lead contact upon request.

## Acknowledgments

This research was conducted using the UK Biobank Resource under application number 694569. The authors thank all UK Biobank participants and the research teams who contributed to the resource. No specific funding was received for this study.

## Author contributions

Y.L. and Z.P., contributed equally to this work; Y.L. and Z.P., conceptualization, methodology, formal analysis, investigation, visualization, and writing – original draft; Z.P., data curation and software; Jing Zhang, investigation, resources, and supervision; H.C., validation, resources, and writing – review & editing; Jingyuan Zhang, conceptualization, supervision, project administration, funding acquisition, and writing – review & editing. All authors read and approved the final manuscript.

## Declaration of interests

The authors declare no competing interests.

## STAR★Methods

### Key resources table

**Table:** 

REAGENT or RESOURCE	SOURCE	IDENTIFIER
**Deposited data**		

UK Biobank cohort data (controlled access)	UK Biobank	https://www.ukbiobank.ac.uk/; Application 694569
Ambient benzene concentrations, 1 km grid, 2006–2020	DEFRA UK-AIR	https://uk-air.defra.gov.uk
NHANES 2017-March 2020	CDC/NCHS	https://www.cdc.gov/nchs/nhanes/
NAFLD GWAS summary statistics	Miao et al.^38^	HGG Adv 2022; 3:100056

**Software and algorithms**		

R, version 4.2.1	R Foundation	https://www.r-project.org/
FastUKB R package	Ke et al.^39^	iMetaMed 2025; 1:e70001
BioAge R package, v2.0.2	Kwon & Belsky et al.^40^	https://github.com/dayoonkwon/BioAge
PLINK	Purcell et al.^41^	https://www.cog-genomics.org/plink/
mediation R package	Tingley et al.^42^	https://CRAN.R-project.org/package=mediation
Machine-learning libraries (scikit-learn, XGBoost, LightGBM, CatBoost)	Open source	https://scikit-learn.org; https://xgboost.ai

### Experimental model and study participant details

#### Study design and participants

This study utilized data from the UK Biobank (UKB), a large-scale prospective cohort study. Since 2006, the UKB has enrolled approximately 500,000 community-dwelling adults aged 37–73 years through 22 assessment centers across the United Kingdom. The UKB systematically collects multidimensional data, including sociodemographic information, lifestyle behaviors, anthropometric measurements, electronic health records, biospecimens, and imaging data. At baseline, all participants completed a touchscreen questionnaire and an in-person interview and provided written informed consent. The UKB resource is available to approved researchers through formal application procedures (https://www.ukbiobank.ac.uk/).

In this study, the baseline was defined as the date of each participant’s first visit to a UK Biobank assessment center. Figure 1 illustrates the overall study design, including participant inclusion and exclusion criteria. Specifically, after excluding individuals with missing benzene exposure data (*N* = 578) and those with incomplete covariate information (*N* = 99,912), a total of 401,472 participants were included in the primary analysis cohort. When integrating genetic data, 332,413 participants with complete genotype, covariate, and genetic principal-component data were included in the PC-adjusted PRS analyses.

Both female and male UK Biobank participants were included. The analytic cohort of 401,472 participants comprised 218,893 women (54.5%) and 182,579 men (45.5%), with a median baseline age of 58 years (interquartile range, 50–63; eligible age range, 37–73 years). Sex (self-reported; UK Biobank field 31) and age were adjusted for in all multivariable models, and the influence of sex on the benzene-NAFLD association was examined in sex-stratified subgroup analyses (Table 4), which showed no significant effect modification by sex (P for interaction >0.05).

#### Ethical statement

This study was conducted using data from the UK Biobank, a large population-based prospective cohort. The UK Biobank received ethical approval from the North West Multi-centre Research Ethics Committee (reference number 11/NW/0382). All participants provided written informed consent prior to enrollment. The present analyses were performed under approved UK Biobank application number 694569. All data were de-identified prior to analysis, and no additional ethical approval was required for this secondary analysis.

#### Consent to participate

All UK Biobank participants provided informed consent to participate at enrollment; therefore, specific consent for this secondary analysis was not required.

### Method details

#### Exposure assessment

The primary exposure variable in this study was the residential benzene concentration. Benzene concentration data were obtained from the long-term monitoring network of the United Kingdom Department for Environment, Food and Rural Affairs (DEFRA), covering annual averages from 2006 to 2020. This monitoring system integrates direct measurements from urban and rural background stations with atmospheric dispersion models to generate nationwide annual benzene concentration estimates on a 1 km × 1 km resolution grid. Model performance was validated against ground-based monitoring observations, showing high correlation and consistency in benzene estimation (coefficient of determination R^2^ ≈ 0.82, root-mean-square error<0.12 μg/m^3^^43^). The dataset is publicly accessible through the DEFRA Air Quality Archive (https://uk-air.defra.gov.uk), and detailed documentation is provided in the supplemental information.

In the UK Biobank, participants’ residential benzene exposure was determined by linking geocoded baseline addresses (easting and northing coordinates; data fields 32223 and 32224) to the corresponding 1 km grid cells. For participants who relocated during follow-up, annual benzene concentrations at each residential address were extracted and weighted by the duration of residence to calculate an individual time-weighted average exposure level.^44^ Specifically, for each participant, the cumulative exposure concentration was estimated as:Ctotal = $\frac{\sum_{i=1}^{n}\left(C_{i}\times T_{i}\right)}{\sum_{i=1}^{n}\left(T_{i}\right)}$,where Ci is the concentration of benzene at the residential address, Ti is the residence duration (years), n is the total number of residential addresses, and i is the number of times participants reside in different places of residence. This approach accounts for variations in exposure intensity across different locations and periods.

#### Ascertainment of outcomes

In the latest expert consensus statement, NAFLD is defined by the International Classification of Diseases, 10th Revision (ICD-10) codes K76.0 and K75.8.^45^ In the UK Biobank study, hospital admission dates and diagnoses were ascertained through linkage with national health databases, including the Hospital Episode Statistics for England, the Scottish Morbidity Record, and the Welsh Patient Episode Database. Participants were followed from the date of baseline assessment until the first diagnosis of NAFLD, loss to follow-up, death, or the end of follow-up-whichever occurred first (October 31, 2022, for England; July 31, 2021, for Scotland; and February 28, 2018, for Wales).

#### Covariates

Based on previous literature, we included covariates that may influence the associations among benzene exposure and NAFLD.^25^^,^^46^ Demographic characteristics comprised age (calculated from the date of birth and baseline assessment date), sex, and ethnicity. Socioeconomic factors included educational attainment, self-reported pre-tax household income at baseline (<£18,000; £18,000–30,999; £31,000–51,999; £52,000–100,000; >£100,000), and the Townsend Deprivation Index, with higher scores indicating greater socioeconomic deprivation. Lifestyle and behavioral factors included smoking status, alcohol consumption, physical activity, and diet quality. Regular physical activity was defined as engaging in ≥150 min of moderate-intensity activity or ≥75 min of vigorous-intensity activity per week, or an equivalent combination of both. The diet score (range 0–7) was derived from the intake of vegetables, fruits, fish, whole grains, refined grains, processed meats, and unprocessed meats, with higher scores reflecting healthier dietary patterns,^47^^,^^48^^,^^49^ (Table S1). Environmental factors included residential distance to the coastline, calculated using geocoded address data. Clinical factors comprised body mass index (BMI, kg/m^2^),^50^ sleep duration, and diagnoses of hypertension, diabetes, and asthma, confirmed by hospitalization records and medication use. Diabetes was defined as a glycated hemoglobin (HbA1c) level >6.5% or the use of glucose-lowering medications. Hypertension was defined based on self-reported history or the use of antihypertensive medications.

#### Polygenic risk score

Genotyping of UKB participants was performed using custom Affymetrix UK BiLEVE Axiom arrays and UK Biobank Axiom arrays, covering approximately 800,000 single-nucleotide polymorphisms (SNPs).^51^ Quality control procedures included removing variants with low sequencing quality, a minor allele frequency (MAF) < 1%, significant deviation from Hardy-Weinberg equilibrium (*p* < 1 × 10^−6^), or a high missingness rate. Genotype data with higher coverage were subsequently imputed using the Haplotype Reference Consortium (HRC) and UK10K reference panels.^52^

To construct a PRS for NAFLD, we selected SNPs that were significantly associated with NAFLD in recent large-scale genome-wide association studies (GWASs) (*p* < 5 × 10^−8^).^38^ Linkage-disequilibrium (LD) pruning and clumping were subsequently performed in PLINK to remove variants in high LD.^41^ Specifically, independent variants were retained within a 1,000-kb window using an LD threshold of r^2^ < 0.10, anchored to the European-ancestry reference panel (Table S2). After excluding variants in high linkage disequilibrium, 31 independent SNPs were retained (Table S3). The PRS was calculated using the following weighted summation formula: *PRS*_*i*_ = $\sum_{k=1}^{K}Xik\beta k$.

In the formula, *PRS*_*i*_ represents the polygenic risk score for individual, X_*ik*_ denotes the number of risk alleles (0, 1, or 2) carried by individual *i* at the *k*-th SNP; *β*_k_ is the corresponding effect size of the *k*-th SNP derived from the GWAS; and K is the total number of SNPs included.^53^ The resulting PRS was standardized using Z-transformation and categorized into low-, intermediate-, and high-genetic-risk groups based on tertiles to facilitate subsequent stratified and interaction analyses.

#### Definition of biological aging

This study comprehensively assessed systemic aging using a multidimensional biological aging framework that incorporated four complementary indices: PhenoAge, Allostatic Load (AL),Accelerated Biological Aging (ABA) and Homeostatic Dysregulation (HD) were calculated according to the standard algorithms from the National Health and Nutrition Examination Survey (NHANES) III (1988–1994), using the R package BioAge (version 2.0.2, commit ID: 1f5c1a7; https://github.com/dayoonkwon/BioAge).^40^ All parameters were implemented following the package’s default settings. All biomarkers were derived from baseline blood samples collected prior to the onset of follow-up and before the occurrence of outcome events, including death.

PhenoAge integrates nine clinical biomarkers-albumin, alkaline phosphatase, creatinine, C-reactive protein, glucose, mean corpuscular volume, red cell distribution width, white blood cell count, and lymphocyte percentage-using an elastic-net-based Gompertz regression model developed by Levine et al.^54^ This index quantifies systemic inflammation and metabolic burden.

HD, derived from the approach proposed by Nakazato et al.^55^, applies the Mahalanobis distance to evaluate the extent to which multiple physiological parameters deviate from a healthy reference distribution, thereby reflecting overall homeostatic imbalance.

AL, a composite indicator of chronic stress and cumulative physiological burden, was calculated from 12 clinical biomarkers: albumin, alkaline phosphatase, C-reactive protein, total cholesterol, creatinine, glycated hemoglobin, systolic blood pressure, blood urea nitrogen, uric acid, lymphocyte percentage, mean cell volume, and white blood cell count (Table S4). Quartile cut-offs for each biomarker were derived from its distribution in the analytic cohort. For all biomarkers except albumin, participants in the highest quartile were classified as “at risk” for that marker; for albumin, in line with previous studies,^46^ participants in the lowest quartile were classified as “at risk”. The AL score was then computed as the number of biomarkers classified as “at risk” divided by 12. AL values therefore range from 0 to 1, representing the proportion of biomarkers for which an individual is at high risk; higher AL values indicate greater cumulative physiological burden and a higher level of systemic metabolic dysregulation.

ABA was defined as the residual of biological age regressed on chronological age, representing the deviation of an individual’s biological age from their expected value given chronological age. A positive residual indicates accelerated aging (biologically older than expected), whereas a negative residual indicates decelerated aging. For consistency and interpretability, the ABA values were standardized before analysis and used as continuous variables in subsequent mediation and interaction models.

### Quantification and statistical analysis

Data extraction and preprocessing were performed using the FastUKB R package, a recently developed tool that streamlines variable selection, data cleaning, and quality control for UK Biobank data.^39^ In this study, multivariable Cox proportional hazards models with age as the underlying timescale were employed to estimate the hazard ratios (HRs) and 95% confidence intervals (CIs) for the association between long-term environmental benzene exposure and the risk of NAFLD after adjusting for potential confounders. The proportional hazards assumption was verified using Schoenfeld residuals. Two progressively adjusted models were constructed. Model A was adjusted for age and sex. Model B was further adjusted for ethnicity, body mass index (BMI), educational attainment, household income, smoking status, alcohol consumption, physical activity, healthy diet score, residential distance from the coastline, Townsend Deprivation Index (TDI), hypertension, diabetes, asthma, and sleep duration, in order to minimize residual confounding. Restricted cubic spline (RCS) models with three knots, placed at the 10th, 50th, and 90th percentiles, were used to assess the dose-response relationship between benzene exposure and NAFLD risk; the number and position of the knots were selected based on the Akaike information criterion (AIC) and the Bayesian information criterion (BIC). When a non-linear association was detected, a two-piecewise Cox proportional-hazards model was further applied to identify the inflection point (K): a grid search was conducted across the benzene-exposure distribution in 0.01 μg/m^3^ increments, with the candidate point yielding the highest log likelihood and the lowest AIC and BIC selected as K. The robustness of the threshold analysis was further evaluated using alternative cut-points(0.250, 0.350, and 0.400 μg/m^3^; Table S21). Segment-specific HRs are reported per interquartile-range (IQR) increment in benzene exposure.To assess potential confounding by co-pollutants, multi-pollutant Cox models were further constructed by adjusting the primary model for NO_2_, PM_2.5,_ and PM_10_ either individually or jointly; changes in the benzene effect estimate across these models were then compared.

To evaluate the joint effects of genetic susceptibility and environmental exposure, a PRS was constructed and participants were categorized into three groups representing low, intermediate, and high genetic risk. A combined stratified analysis was performed to examine the cumulative effects of benzene exposure and PRS on the risk of NAFLD, using the low-exposure and low-genetic-risk group as the reference. Potential interactions between benzene exposure and genetic risk were further assessed under both multiplicative and additive models. Additive interactions were quantified using the relative excess risk due to interaction (RERI), the attributable proportion (AP), and the synergy index (SI), with corresponding 95% CIs estimated using the delta method.All PRS-related models were additionally adjusted for the first 10 genetic principal components (UK Biobank field 22009) to control for population stratification.

Mediation analysis was performed to evaluate whether biomarkers of biological aging mediated the association between benzene exposure and NAFLD risk. The proportion mediated and its 95% confidence interval (CI) were estimated on the hazard ratio (HR) scale using 5,000 bootstrap resamples.^42^ Four biological aging indices-PhenoAge, HD, AL, and ABA-were analyzed to assess their individual and relative contributions to the association between benzene exposure and NAFLD.To further clarify the effect decomposition, the total effect, average mediation effect, average direct effect, and proportion mediated were reported, along with estimates of the natural indirect effect (NIE) and natural direct effect (NDE).

A multi-model machine-learning framework was used to evaluate the predictive performance of benzene exposure and covariates for NAFLD risk. The full cohort was randomly split into training and validation sets in a 7:3 ratio, stratified by NAFLD event status to ensure identical event rates in the two sets. Ten algorithms were trained on the training set: logistic regression, support vector machine (SVM), neural network, gradient boosting machine (GBM), random forest, extreme gradient boosting (XGBoost), LightGBM, AdaBoost, CatBoost, and k-nearest neighbors (KNN). Hyperparameters were tuned by stratified 5-fold cross-validation within the training set; given the low event rate of NAFLD, class weights inversely proportional to class frequency (class_weight = “balanced”) were applied during training to mitigate class imbalance. Model performance was evaluated on the held-out validation set using the area under the receiver-operating-characteristic curve (ROC AUC), the area under the precision-recall curve (PR AUC), accuracy, sensitivity, specificity, balanced accuracy, precision, and F1 score; the classification threshold was determined in the validation set and used to compute threshold-specific classification metrics. The model with the best combined ROC AUC and accuracy was selected as the final model. Shapley additive explanations (SHAP) were used to quantify the contribution and directionality of each predictor. A nomogram was further constructed from the final model, and its discrimination and calibration in the validation set were assessed using ROC and calibration curves.

Several sensitivity analyses were performed to evaluate the robustness of our findings. First, Fine-Gray subdistribution hazard models were fitted with death as a competing event to assess the impact of competing risks on the primary results. Second, NAFLD was redefined by combining the hepatic steatosis index (HSI) with ICD-10 codes K76.0 and K75.8 to verify the consistency of the outcome definition; case counts and the overlap across alternative NAFLD definitions were also reported. Third, the analysis was restricted to participants who had lived at the same residential address for at least 10 years, to reduce exposure misclassification arising from address changes. Fourth, the primary analysis was repeated using alternative benzene-exposure thresholds to examine the influence of the exposure-categorisation criteria on the results. Fifth, multi-pollutant models were constructed to evaluate the independence of the benzene-NAFLD association after additional adjustment for NO_2_, PM_2.5_, and PM_10_, either individually or jointly. Finally, to reduce potential reverse causation and address the temporal alignment of exposure, mediator, and outcome, lag analyses were performed in which participants who developed NAFLD within the first 1 or 2 years of follow-up were excluded; in addition, the mediation analysis was repeated using the annual benzene concentration in the baseline-assessment year as an alternative exposure metric, and was further restricted to participants who had lived at the same residential address for ≥10 years, to test the robustness of the mediation results.

Finally, to provide external-consistency evidence in an independent population, we examined the association between urinary trans,trans-muconic acid (TTMA), a biomarker of benzene exposure, and HSI-defined hepatic steatosis in NHANES (2017-March 2020; *n* = 1,681), using survey-weighted logistic regression that accounted for the complex survey design.

## Supplemental information

Document S1. Tables S1–S5, S7–S12, S14–S18, S20–S27, and S29

Table S6. Distribution of long-term benzene exposure and key baseline covariates across polygenic risk score tertiles

Table S13. Mediation of the benzene-NAFLD association by four biological-aging measures, showing natural direct and indirect effects and mediated proportions

Table S19. Baseline characteristics of the 368,819 UK Biobank participants included in the multi-pollutant sensitivity analysis

Table S28. Baseline characteristics of the NHANES external-validation sample across urinary TTMA quartiles
