# Supplementary material for: Long-term ambient benzene exposure increases incident NAFLD risk through accelerated biological aging
Source: iScience. 2026 Sep 30;29(10):117035. doi: 10.1016/j.isci.2026.117035 (PMC13657151; doi:10.1016/j.isci.2026.117035)
Supplement: Supplementary file 1 [file mmc1.pdf]

iScience, Volume *29*

Supplemental information

Long-term ambient benzene exposure increases incident NAFLD risk through accelerated biological aging

Yaxiong Li, Ziwei Peng, Jing Zhang, Hong Cao, and Jingyuan Zhang

Table of contents

Supplementary Table 1. Components of diet score in the UK Biobank. 3

Supplementary Table 2. PRS construction, LD pruning, and population stratification control. 4

Supplementary Table 3. SNPs used to construct the polygenic risk score for NAFLD 6

Supplementary Table 4. Components of biological ages according to NAFLD cases 8

Supplementary Table 5. Risk of NAFLD according to long-term exposure to benzene based on different genetic risk categories. 10

Supplementary Table 7. Association between long-term benzene exposure and NAFLD risk stratified by genetic risk categories after adjustment for genetic principal components. 10

Supplementary Table 8. Additive interaction between long-term benzene exposure and genetic risk on the risk of NAFLD 11

Supplementary Table 9. The association between polygenic risk score and incident NAFLD in the European ancestry subgroup. 12

Supplementary Table 10. Association between long-term benzene exposure and NAFLD risk stratified by genetic risk categories in the European ancestry subgroup. 13

Supplementary Table 11. Joint association of long-term benzene exposure and genetic risk with incident NAFLD in the European ancestry subgroup. 14

Supplementary Table 12. Additive interaction between long-term benzene exposure and genetic risk on the risk of NAFLD in the European ancestry subgroup. 14

Supplementary Table 14. Sensitivity analysis for the association between long-term benzene exposure and NAFLD risk accounting for death as a competing event.[abc] 15

Supplementary Table 15. Case counts and overlap across alternative NAFLD definitions based on ICD-10 codes, HSI, and the combined HSI+ICD definition 16

Supplementary Table 16. Sensitivity analysis for the association between long-term benzene exposure and NAFLD risk defined by HSI and ICD-10 codes [abc]. 17

Supplementary Table 17. The associations between long-term benzene exposure and NAFLD risk by restricting analyses to those living at the same address for ≥10 years [abc] 18

Supplementary Table 18. Sensitivity analysis for the association between long-term benzene exposure and NAFLD risk according to different exposure thresholds abc 19

Supplementary Table 20. Associations between Long-Term Benzene Exposure and the Risk of NAFLD in Single- and Multi-Pollutant Models. 20

Supplementary Table 21. Threshold associations of benzene with incident NAFLD using alternative candidate cutpoints. 21

Supplementary Table 22. Sensitivity analyses addressing temporal alignment between benzene exposure and incident NAFLD. 22

Supplementary Table 23. Sensitivity analyses of mediation results addressing temporal ordering of benzene exposure, biological aging biomarkers, and incident NAFLD. 24

Supplementary Table 24. Data split for the machine-learning analysis in the ICD-defined NAFLD cohort. 26

Supplementary Table 25. Validation discrimination metrics for 10 machine-learning models. 26

Supplementary Table 26. Threshold-specific classification metrics on the validation set. 27

Supplementary Table 27. Sample selection flow for the urinary TTMA-HSI analytic sample 28

Supplementary Table 29. Adjusted odds ratios for HSI-defined hepatic steatosis by urinary TTMA 29

Supplementary Table 1. Components of diet score in the UK Biobank.

Diet component	Intake goal	Field ID in UK Biobank	Amount per serving
Fruits	≥3 servings/day	1309 (pieces fresh fruit/day);<br>1319 (pieces dried fruit/day)	1309 - 1 piece;<br>1319 - 5 pieces
Vegetable	≥3 servings/day	1289 (tablespoons cooked vegetables/day);<br>1299 (salad/raw vegetables/day)	3 heaped tablespoons
Fish	≥2 servings/week	1329 (oily fish/week);<br>1339 (non-oily fish/week)	Once/week
Whole grains	≥3 servings/day	1438, 1448 (wholemeal or wholegrain bread slices/week);<br>1458, 1468 (bran/oat/muesli cereal bowls/week)	1438/1448 - 1 slice/day;<br>1458/1468 - 1 bowl/day

Refined grains	≤2 servings/day	1438, 1448 (white, brown, other bread slices/week); 1458, 1468 (biscuit, other cereals/week)	1438/1448 - 1 slice/day; 1458/1468 - 1 bowl/day
Processed meats	≤1 servings/week	1349 (processed meat/week or daily); 3680 (age when last ate meat)	1349 - 1 piece/day; 3680 - 0 pieces/day if indicated having never eaten meat
Unprocessed meats	≤2 servings/week	1359 (poultry/week or day); 1369 (beef/week or day); 1379 (lamb or mutton/week or day); 1389 (pork/week or day); 3680 (age when last ate meat)	1359-1389 - once/week; 3680 - 0 pieces/day if indicated having never eaten meat

Supplementary Table 2. PRS construction, LD pruning, and population stratification control.

Item	Description
Genetic analysis sample	A total of 332,413 participants with complete genotype data were included in the PRS analyses.
Source GWAS	Previously published large-scale genome-wide association study of NAFLD susceptibility loci[1].
Variant inclusion threshold	Genome-wide significant variants associated with NAFLD ($P < 5\times10^{-8}$) were eligible for PRS construction.

LD pruning / clumping software	PLINK was used for LD pruning / clumping.
LD pruning parameters	Independent variants were retained using a 1,000-kb window and an LD threshold of $r^2 < 0.10$.
Reference panel for LD	1000 Genomes Project Phase 3 European-ancestry reference panel.
Number of retained SNPs	A total of 31 independent SNPs were retained after LD pruning and used for PRS construction.
PRS calculation	The PRS was calculated as the weighted sum of effect-allele dosage multiplied by the published beta coefficient for each SNP.
Standardization	The continuous PRS was z-standardized before subsequent analyses.
Risk grouping	The standardized PRS was categorized into low, intermediate, and high genetic-risk groups according to tertiles.
Genetic principal components	Genetic principal components were obtained from UK Biobank field 22009. The first 10 components (PC1-PC10) were extracted and used to control population stratification.
Population stratification control	PC1-PC10 were included as covariates in all PRS-related Cox proportional hazards models, including the PRS main-effect analysis, benzene-NAFLD analyses stratified by PRS group, joint/cumulative risk analyses of benzene exposure and genetic risk, and additive interaction analyses.
Consistency across PRS analyses	The same PC1-PC10 adjustment strategy was applied across the full-cohort PRS analyses. The descriptive

	table of benzene exposure and key covariates across PRS tertiles was restricted to the same PC-adjusted analytic population.
European-ancestry sensitivity analysis	As a secondary analysis, PRS analyses were repeated in the genetically defined European-ancestry subgroup using UK Biobank genetic ethnic grouping field 22006, with PC1-PC10 retained in the models.

Notes: GWAS, genome-wide association study; LD, linkage disequilibrium; NAFLD, nonalcoholic fatty liver disease; PC, principal component; PRS, polygenic risk score; SNP, single-nucleotide polymorphism.

Supplementary Table 3. SNPs used to construct the polygenic risk score for NAFLD

rsID	Chr	Position	Locus	EA	OA	EAF	OR (95% CI)	Beta weight	Effect direction
RS1015445	11	17847126	KCNC1,SERGEF	C	T	0.348	0.89 (0.84-0.93)	-0.121825	Risk-decreasing
RS10810259	9	14831866	FREM1	G	C	0.462	1.13 (1.08-1.18)	0.120829	Risk-increasing
RS112875651	8	125494452	RP11-136O12.2	A	G	0.386	0.83 (0.79-0.87)	-0.184290	Risk-decreasing
RS113092519	8	145075880	C8orf33	C	G	0.034	0.60 (0.51-0.70)	-0.513703	Risk-decreasing
RS12444598	16	80369700	RP11-525K10.3	G	A	0.783	0.87 (0.83-0.92)	-0.133847	Risk-decreasing
RS12488765	3	24660179	THRB-AS1	G	A	0.248	0.87 (0.83-0.92)	-0.135198	Risk-decreasing
RS12614574	2	189633406	RP11-88L20.1	T	C	0.039	0.73 (0.64-0.83)	-0.315667	Risk-decreasing

RS149107791	8	96577208	SDC2	G	A	0.036	0.72 (0.62-0.82)	-0.332432	Risk-decreasing
RS184939711	4	99769602	DAPP1	A	G	0.035	1.35 (1.20-1.52)	0.299988	Risk-increasing
RS199616596	9	61413065	CNTNAP3C	C	T	0.327	0.86 (0.81-0.92)	-0.147709	Risk-decreasing
RS2267595	22	44031371	PARVB	C	T	0.073	1.37 (1.26-1.49)	0.315841	Risk-increasing
RS2440807	7	138154717	AC083867.4	C	T	0.665	0.87 (0.83-0.92)	-0.136476	Risk-decreasing
RS2745359	6	127060811	VIM2P	C	T	0.083	1.22 (1.13-1.32)	0.199703	Risk-increasing
RS2803609	10	112159366	GPAM	G	A	0.684	0.89 (0.84-0.93)	-0.119365	Risk-decreasing
RS28429148	16	53764407	FTO	A	G	0.471	1.12 (1.07-1.17)	0.112239	Risk-increasing
RS28636836	4	87310713	HSD17B13	T	C	0.216	0.84 (0.79-0.89)	-0.179178	Risk-decreasing
RS3747207	22	43928975	PNPLA3	A	G	0.227	1.60 (1.52-1.68)	0.468469	Risk-increasing
RS5764468	22	44022567	PARVB	C	T	0.534	0.89 (0.85-0.93)	-0.119803	Risk-decreasing
RS60549462	19	3379997	NFIC	G	T	0.036	0.69 (0.60-0.80)	-0.366999	Risk-decreasing
RS6543124	2	102370999	IL18R1	A	T	0.408	1.13 (1.08-1.18)	0.121601	Risk-increasing
RS693190	3	145466667	LARP7P4	G	T	0.019	1.46 (1.25-1.70)	0.379623	Risk-increasing
RS7032795	9	114372665	AKNA	C	T	0.477	0.90 (0.85-0.94)	-0.109942	Risk-decreasing
RS73027058	3	12890204	LINC02022	G	C	0.109	0.80 (0.74-0.87)	-0.220803	Risk-decreasing
RS73115877	4	14919559	CPEB2-DT	C	T	0.092	0.80 (0.73-0.87)	-0.222093	Risk-decreasing

RS77917801	16	80589731	CDYL2	T	G	0.067	0.79 (0.71-0.87)	-0.238361	Risk-decreasing
RS780093	2	27519736	GCKR	C	T	0.648	0.89 (0.85-0.93)	-0.119276	Risk-decreasing
RS78572354	10	112254502	TECTB	G	T	0.166	0.85 (0.79-0.91)	-0.164133	Risk-decreasing
RS8023151	14	95599206	LINC02318	C	T	0.306	0.88 (0.84-0.93)	-0.126479	Risk-decreasing
RS8100204	19	19282905	SUGP1	A	G	0.159	1.36 (1.28-1.45)	0.309157	Risk-increasing
RS8106159	19	20025569	ZNF682	T	C	0.778	0.88 (0.83-0.93)	-0.130517	Risk-decreasing
RS929092	22	43921204	PNPLA3	G	A	0.445	1.18 (1.12-1.23)	0.161663	Risk-increasing

Abbreviations: Chr, Chromosome; CI, confidence interval; EA, effect allele; EAF, effect allele frequency; GWAS, genome-wide association study; OR odds ratio; SNP, single nucleotide polymorphism.

Supplementary Table 4. Components of biological ages according to NAFLD cases

	UKB field ID	NAFLD cases	
Indicator		No (N = 395,624)	Yes (N = 5,848)
Albumin, g/L	30600 (g/L)	39.35(39.34,39.37)	38.89(38.78,39.00)
Alkaline phosphatase, U/L	30610 (U/L)	83.21(83.14,83.27)	92.67(92.01,93.32)
C-reactive protein, mg/dL	30710 (mg/L)	0.25(0.25,0.25)	0.42(0.40,0.43)

Total cholesterol, mmol/L	30690 (mmol/L)	4.64(4.63,4.64)	4.40(4.37,4.43)
Creatinine, umol/L	30700 (umol/L)	67.34(67.28,67.40)	69.05(68.57,69.52)
Glycated hemoglobin, %	30750 (mmol/mol)	5.45(5.45,5.45)	5.75(5.73,5.77)
Systolic blood pressure, mmHg	4080 (mmHg)	137.82(137.77,137.88)	140.53(140.11,140.95)
Blood urea nitrogen, mmol/L	30670 (mmol/L)	5.41(5.40,5.41)	5.45(5.41,5.48)
Uric acid, umol/L	30880 (umol/L)	308.90(308.67,309.13)	342.79(340.80,344.78)
Lymphocyte percent, %	30180 (%)	28.88(28.86,28.90)	28.89(28.72,29.06)
Mean cell volume, fL	30040 (fL)	91.10(91.08,91.11)	91.06(90.94,91.19)
White blood cell count, 10^9 cells/L	30000 (10^9 cells/L)	6.86(6.85,6.86)	7.41(7.36,7.45)

Notes: Values are mean (95% CI) of each biomarker measured at baseline, stratified by incident NAFLD status. UK Biobank field IDs are listed for reference; units are reported as commonly used in clinical practice (HbA1c is shown in % rather than mmol/mol for ease of interpretation). The 12 biomarkers shown were used to compute Phenotypic Age (PhenoAge), Homeostatic Dysregulation (HD), and Allostatic Load (AL); details of the algorithms are provided in Methods Section 2.6. Abbreviations: CI, confidence interval; NAFLD, nonalcoholic fatty liver disease; UKB, UK Biobank.

Supplementary Table 5. Risk of NAFLD according to long-term exposure to benzene based on different genetic risk categories.

**Genetic Risk Category**	**Benzene Exposure Level**	**Events**	**Total / Person years**	**Adjusted HR (95% CI)**
**Low Genetic Risk**	Low Benzene exposure	690	55,875 / 751,310.674	Ref
	High Benzene exposure	742	55,004 / 711,155.468	1.04 (0.94-1.15)
**Intermediate Genetic Risk**	Low Benzene exposure	748	54,890 / 737,859.688	1.12 (1.01-1.24)
	High Benzene exposure	874	55,874 / 722,042.682	1.25 (1.13-1.38)
**High Genetic Risk**	Low Benzene exposure	964	54,303 / 728,769.921	1.42 (1.28-1.56)
	High Benzene exposure	1,086	56,467 / 727,405.118	1.73 (1.56-1.91)

The low genetic risk and low benzene exposure group was used as the reference. The model was adjusted for age, sex, ethnicity, BMI, education level, household income, smoking status, alcohol drinking status, healthy diet score, physical activity, distance from residence to the coast, TDI, hypertension, diabetes, asthma, sleep duration, and the first 10 genetic principal components.

Supplementary Table 7. Association between long-term benzene exposure and NAFLD risk stratified by genetic risk categories after adjustment for genetic principal components.

Genetic Risk Category	Benzene Exposure Level	Events	Total / Person years	HR (95% CI)	P value
Low Genetic Risk	Low Benzene exposure	690	55,875 / 751,310.674	Ref	

	High Benzene exposure	742	55,004 / 711,155.468	1.04 (0.94-1.15)	0.418
Intermediate Genetic Risk	Low Benzene exposure	748	54,890 / 737,859.688	Ref	
	High Benzene exposure	874	55,874 / 722,042.682	1.12 (1.01-1.24)	0.035
High Genetic Risk	Low Benzene exposure	964	54,303 / 728,769.921	Ref	
	High Benzene exposure	1,086	56,467 / 727,405.118	1.22 (1.11-1.34)	<0.001

Hazard ratios compare high versus low benzene exposure within each genetic risk category. The model was adjusted for age, sex, ethnicity, BMI, education level, household income, smoking status, alcohol drinking status, healthy diet score, physical activity, distance from residence to the coast, TDI, hypertension, diabetes, asthma, sleep duration, and the first 10 genetic principal components.

Supplementary Table 8. Additive interaction between long-term benzene exposure and genetic risk on the risk of NAFLD

Exposure × Genetic risk	Model a			Model b		
	RERI (95% CI)	AP (95% CI)	SI (95% CI)	RERI (95% CI)	AP (95% CI)	SI (95% CI)
High Benzene × High Genetic Risk	0.30 (0.09, 0.51)	0.18 (0.06, 0.30)	1.52 (1.12, 2.06)	0.27 (0.06, 0.48)	0.16 (0.04, 0.28)	1.46 (1.08, 1.98)

Model a was adjusted for age, sex, BMI, and the first 10 genetic principal components. Model b was adjusted for age, sex, ethnicity, BMI, education

level, household income, smoking status, alcohol drinking status, healthy diet score, physical activity, distance from residence to the coast, TDI, hypertension, diabetes, asthma, sleep duration, and the first 10 genetic principal components.

Supplementary Table 9. The association between polygenic risk score and incident NAFLD in the European ancestry subgroup.

			Model a		Model b		Model c	
Genetic Risk Category	Events	Total/Person years	HR (95% CI)	P value	HR (95% CI)	P value	HR (95% CI)	P value
Polygenic risk score	4,218	275,932/3,635,389.770	1.36 (1.28-1.43)	1.97×10^{-27}	1.35 (1.27-1.42)	7.68×10^{-26}	1.35 (1.27-1.42)	8.13×10^{-26}
Low Genetic Risk	1,223	93,852/1,237,553.225	ref		ref		ref	
Intermediate Genetic Risk	1,315	91,865/1,211,527.534	1.10 (1.02-1.19)	0.018	1.07 (0.99-1.16)	0.073	1.07 (0.99-1.16)	0.072
High Genetic Risk	1,680	90,215/1,186,309.011	1.44 (1.33-1.55)	5.98×10^{-22}	1.42 (1.31-1.52)	2.51×10^{-20}	1.42 (1.32-1.52)	2.20×10^{-20}

The European ancestry subgroup was defined using UK Biobank genetic ethnic grouping field 22006. All analyses were additionally adjusted for the first 10 genetic principal components. Model a was adjusted for age, sex, and the first 10 genetic principal components. Model b was adjusted

for age, sex, BMI, education level, household income, smoking status, alcohol drinking status, healthy diet score, physical activity, distance from residence to the coast, TDI, hypertension, diabetes, asthma, sleep duration, and the first 10 genetic principal components. Model c was further adjusted for benzene exposure. Abbreviations: BMI, body mass index; CI, confidence interval; HR, hazard ratio; NAFLD, non-alcoholic fatty liver disease.

Supplementary Table 10. Association between long-term benzene exposure and NAFLD risk stratified by genetic risk categories in the European ancestry subgroup.

Genetic Risk Category	Benzene Exposure Level	Events	Total / Person years	HR (95% CI)	P value
Low Genetic Risk	Low Benzene exposure	633	49,997 / 671,274.252	Ref	
	High Benzene exposure	590	43,855 / 566,278.973	1.02 (0.91-1.14)	0.726
Intermediate Genetic Risk	Low Benzene exposure	666	48,667 / 653,656.134	Ref	
	High Benzene exposure	649	43,198 / 557,871.400	1.13 (1.01-1.27)	0.037
High Genetic Risk	Low Benzene exposure	847	47,857 / 641,479.825	Ref	
	High Benzene exposure	833	42,358 / 544,829.186	1.23 (1.10-1.37)	<0.001

The European ancestry subgroup was defined using UK Biobank genetic ethnic grouping field 22006. All analyses were additionally adjusted for the first 10 genetic principal components. Hazard ratios compare high versus low benzene exposure within each genetic risk category. The model

was adjusted as in Model b.

Supplementary Table 11. Joint association of long-term benzene exposure and genetic risk with incident NAFLD in the European ancestry subgroup.

Genetic Risk Category	Benzene Exposure Level	Events	Total / Person years	Adjusted HR (95% CI)
Low Genetic Risk	Low Benzene exposure	633	49,997 / 671,274.252	Ref
	High Benzene exposure	590	43,855 / 566,278.973	1.02 (0.91-1.14)
Intermediate Genetic Risk	Low Benzene exposure	666	48,667 / 653,656.134	1.07 (0.96-1.19)
	High Benzene exposure	649	43,198 / 557,871.400	1.21 (1.08-1.35)
High Genetic Risk	Low Benzene exposure	847	47,857 / 641,479.825	1.38 (1.24-1.53)
	High Benzene exposure	833	42,358 / 544,829.186	1.70 (1.52-1.90)

The European ancestry subgroup was defined using UK Biobank genetic ethnic grouping field 22006. All analyses were additionally adjusted for the first 10 genetic principal components. The model was adjusted as in Model b.

Supplementary Table 12. Additive interaction between long-term benzene exposure and genetic risk on the risk of NAFLD in the European ancestry subgroup.

Exposure × Genetic	Model a	Model a	Model a	Model b	Model b	Model b

risk						
	RERI (95% CI)	AP (95% CI)	SI (95% CI)	RERI (95% CI)	AP (95% CI)	SI (95% CI)
High Benzene × High Genetic Risk	0.34 (0.10, 0.58)	0.19 (0.06, 0.33)	1.79 (1.18, 2.71)	0.30 (0.08, 0.52)	0.18 (0.05, 0.31)	1.75 (1.14, 2.68)

The European ancestry subgroup was defined using UK Biobank genetic ethnic grouping field 22006. All analyses were additionally adjusted for the first 10 genetic principal components. Model a and Model b were adjusted as described above. RERI indicates the relative excess risk due to interaction; AP represents the attributable proportion due to interaction; SI indicates the synergy index. Values >0 for RERI/AP or >1 for SI suggest positive additive interaction.

Supplementary Table 14. Sensitivity analysis for the association between long-term benzene exposure and NAFLD risk accounting for death as a competing event.[abc]

				SHR (95% CI)		
**Pollution**	**Increase**	**Events**	**Total/person-years**	**Model a**	**Model b**	***P* for trend**
Benzene, μg/m³	per IQR increment	5,848	401,472 / 5,322,586.65	1.26 (1.22-1.30)	1.24 (1.20-1.28)	3.1×10^{-38}
	Q1 (ref)	1,164	99,206 / 1,363,073.12	ref	ref	

	Q2 (0.353-0.43]	1,577	98,789 / 1,347,240.82	1.35 (1.25, 1.46)	1.32 (1.23, 1.42)
	Q3 (0.43-0.504]	1,438	98,938 / 1,336,796.63	1.26 (1.16, 1.37)	1.20 (1.11, 1.30)
	Q4 (0.504-1.13]	1,669	98,691 / 1,275,476.08	1.61 (1.49, 1.74)	1.49 (1.38, 1.61)

[a] Model a: Cox proportional hazards model adjusted for age and sex. [b] Model b: Cox proportional hazards model adjusted for age, sex, ethnicity, BMI, education level, smoking status, alcohol drinking status, physical activity, household income, healthy diet score, distance from residence to the coast, TDI, hypertension, diabetes, asthma, and sleep duration. [c] Abbreviations: SHR, subdistribution hazard ratio; CI, confidence interval; NAFLD, nonalcoholic fatty liver disease.

Supplementary Table 15. Case counts and overlap across alternative NAFLD definitions based on ICD-10 codes, HSI, and the combined HSI+ICD definition

Definition	Cases, n	% of analytic cohort	Status / source
ICD-10-defined incident NAFLD	5848	1.46%	Primary outcome in the analytic cohort (N=401472)
HSI-defined NAFLD (HSI > 36)	155535	38.74%	Derived from baseline HSI using the HSI > 36 threshold
Combined HSI+ICD definition	157215	39.16%	Union of ICD positivity or HSI > 36

ICD only	1680	0.42%	ICD positive but HSI≤36 or HSI unavailable
HSI only	151367	37.70%	HSI > 36 without ICD-coded NAFLD
Both ICD and HSI > 36	4168	1.04%	Overlap between ICD- and HSI-based definitions
Indeterminate for combined HSI+ICD	25252	6.29%	ICD-negative participants with unavailable HSI

Notes: Counts were calculated within the analytic cohort of 401,472 participants. ICD-10–defined incident NAFLD (codes K76.0 and K75.8) was the primary outcome. HSI-defined NAFLD was based on a baseline HSI > 36. The combined HSI + ICD-10 definition classified participants as having NAFLD if they were either ICD-10–positive or had a HSI > 36. Abbreviations: HSI, hepatic steatosis index; ICD-10, International Statistical Classification of Diseases and Related Health Problems, 10th Revision; NAFLD, nonalcoholic fatty liver disease.

Supplementary Table 16. Sensitivity analysis for the association between long-term benzene exposure and NAFLD risk defined by HSI and ICD-10 codes [abc].

				HR (95% CI)		
**Pollution**	**Increase**	**Events**	**Total/person-years**	**Model a**	**Model b**	**P for trend**
Benzene, μg/m³	per IQR increment	157,215	376,220/4,986,334	1.38 (1.37-1.39)	1.36 (1.35-1.37)	1.73×10^{-27}

	Q1 (ref)	38,599	94,056/1,277,918	ref	ref	
	Q2 (0.352,0.428]	39,374	94,054/1,262,885	1.11 (1.09,1.12)	1.11 (1.09,1.12)	
	Q3 (0.428,0.503]	39,942	94,055/1,249,434	1.12 (1.10,1.14)	1.10 (1.08,1.11)	
	Q4 (0.503,1.13]	39,300	94,055/1,196,097	1.83 (1.81,1.86)	1.74 (1.71,1.76)	

[a] Model a: Cox proportional hazards model adjusted for age and sex. [b] Model b: Cox proportional hazards model adjusted for age, sex, ethnicity, BMI, education level, smoking status, alcohol drinking status, physical activity, household income, healthy diet score, distance from residence to the coast, TDI, hypertension, diabetes, asthma, and sleep duration. [c] Abbreviations: NAFLD, nonalcoholic fatty liver disease; BMI, body mass index; CI, confidence interval; HR, hazard ratio; Q, quartile; TDI, Townsend Deprivation Index; μg/m³, micrograms per cubic meter.

Supplementary Table 17. The associations between long-term benzene exposure and NAFLD risk by restricting analyses to those living at the same address for ≥10 years [abc].

				HR (95% CI)		
**Pollution**	**Increase**	**Events**	**Total/person-years**	**Model a**	**Model b**	**P for trend**
Benzene, μg/m³	per IQR increment	3,893	272,135/3,600,970	1.30 (1.25-1.36)	1.28 (1.22-1.33)	7.86×10^{-31}
	Q1 (ref)	748	68,034/923,639.7	ref	ref	
	Q2 (0.353,0.43]	1,077	68,034/911,102.3	1.47 (1.34,1.61)	1.45 (1.32,1.59)	
	Q3 (0.43,0.503]	948	68,033/904,086.1	1.31 (1.19,1.44)	1.24 (1.13,1.37)	

	Q4 (0.503,1.13]	1,120	68,034/862,141.7	1.71 (1.56,1.88)	1.59 (1.44,1.74)

[a] Model a: Cox proportional hazards model adjusted for age and sex. [b] Model b: Cox proportional hazards model adjusted for age, sex, ethnicity, BMI, education level, smoking status, alcohol drinking status, physical activity, household income, healthy diet score, distance from residence to the coast, TDI, hypertension, diabetes, asthma, and sleep duration. [c] Abbreviations: NAFLD, nonalcoholic fatty liver disease; BMI, body mass index; CI, confidence interval; HR, hazard ratio; Q, quartile; TDI, Townsend Deprivation Index; μg/m³, micrograms per cubic meter.

Supplementary Table 18. Sensitivity analysis for the association between long-term benzene exposure and NAFLD risk according to different exposure thresholds abc

				HR (95% CI)		
Pollution	Increase	Events	Total/person-years	Model a	Model b	P for trend
Benzene, μg/m³	per IQR increment	5,848	401,472/5,322,587	1.27 (1.23-1.32)	1.26 (1.21-1.30)	2.27×10^{-40}
	≤0.4 μg/m³	2,055	159,075 / 2,153,183.090	ref	ref	
	≤0.6 μg/m³	5,190	365,748 / 4,878,632.052	1.26 (1.21-1.32)	1.10 (1.05-1.15)	
	≤0.8 μg/m³	5,789	399,622 / 5,300,705.984	1.26 (1.22-1.30)	1.16 (1.12-1.20)	
	No threshold	5,848	401,472 / 5,322,586.649	1.27 (1.23-1.32)	1.19 (1.14-1.23)	

a Model a: Cox proportional hazards model adjusted for age and sex. b Model b: Cox proportional hazards model adjusted for age, sex, ethnicity, BMI, education level, smoking status, alcohol drinking status, physical activity, household income, healthy diet score, distance from residence to the coast, TDI, hypertension, diabetes, asthma, and sleep duration. c Abbreviations: NAFLD, nonalcoholic fatty liver disease; BMI, body mass

index; CI, confidence interval; HR, hazard ratio; Q, quartile; TDI, Townsend Deprivation Index; μg/m³, micrograms per cubic meter.

Supplementary Table 20. Associations between Long-Term Benzene Exposure and the Risk of NAFLD in Single- and Multi-Pollutant Models.

Analysis	Co-pollutant adjustment	N / Events	Benzene HR (95% CI)	P value
Single-pollutant model	None	368,819 / 5,633	1.24 (1.19, 1.29)	<0.001
Two-pollutant model	+ NO_2	368,819 / 5,633	1.17 (1.12, 1.23)	<0.001
Two-pollutant model	+ $PM_{2.5}$	368,819 / 5,633	1.20 (1.15, 1.26)	<0.001
Two-pollutant model	+ PM_{10}	368,819 / 5,633	1.22 (1.16, 1.28)	<0.001
Three-pollutant model	+ NO_2 + $PM_{2.5}$	368,819 / 5,633	1.15 (1.09, 1.21)	<0.001
Four-pollutant model	+ NO_2 + $PM_{2.5}$ + PM_{10}	368,819 / 5,633	1.13 (1.06, 1.20)	<0.001

Notes: Hazard ratios (HRs) and 95% confidence intervals (CIs) were estimated per IQR increment in benzene concentration using Cox proportional hazards models. All models were restricted to participants with complete benzene and co-pollutant exposure data and were adjusted for age, sex, ethnicity, BMI, education level, smoking status, alcohol consumption, physical activity, household income, healthy diet score, residential distance to the coast, Townsend Deprivation Index, hypertension, diabetes, asthma, and sleep duration. NAFLD, nonalcoholic fatty liver disease; BMI, body mass index; CI, confidence interval; HR, hazard ratio; IQR, interquartile range; TDI, Townsend Deprivation Index.

Supplementary Table 21. Threshold associations of benzene with incident NAFLD using alternative candidate cutpoints.

NAFLD	Participants (%)	Events	Person-years	HR (95% CI)	P value
Continuous, per IQR	401,472 (100.0)	5,848	5,322,586.649	1.26 (1.21-1.30)	2.27×10^-40
Inflection point (K)	0.250 μg/m³				
<K	18,631 (4.6)	148	260,167.795	1.05 (0.94-1.17)	0.392
⩾K	382,841 (95.4)	5,700	5,062,418.855	1.27 (1.21-1.33)	<0.001
Likelihood ratio test for threshold model					0.018
Inflection point (K)	0.350 μg/m³				
<K	97,762 (24.4)	1,127	1,327,965.490	1.08 (1.04-1.12)	<0.001
⩾K	303,710 (75.6)	4,721	3,994,621.159	1.32 (1.25-1.40)	<0.001
Likelihood ratio test for threshold model					0.006
Inflection point (K)	0.400 μg/m³				

<K	159,075 (39.6)	2,055	2,153,183.090	1.10 (1.05-1.15)	<0.001
⩾K	242,397 (60.4)	3,793	3,169,403.559	1.30 (1.22-1.38)	<0.001
Likelihood ratio test for threshold model					0.024

Notes: Hazard ratios (HRs) and 95% confidence intervals (CIs) were estimated using Cox proportional hazards models. The continuous estimate was reported per IQR increment in benzene concentration. Segment-specific HRs below and above each candidate cutpoint were estimated using two-piecewise Cox proportional hazards regression models and are presented per IQR increase in benzene concentration. The likelihood ratio test compared each two-piecewise model with the single-line Cox model. NAFLD, nonalcoholic fatty liver disease; CI, confidence interval; HR, hazard ratio; IQR, interquartile range.

Supplementary Table 22. Sensitivity analyses addressing temporal alignment between benzene exposure and incident NAFLD.

Analysis	Exposure metric or restriction	Participants	NAFLD events	Person-years	HR (95% CI)	P value
Primary analysis	Time-weighted average benzene exposure during follow-up	401,472	5,848	5,322,586.649	1.26 (1.21-1.30)	2.27×10^{-40}
Baseline-year exposure	Annual benzene concentration in the baseline assessment year	401,472	5,848	5,322,586.649	1.22 (1.17-1.27)	4.81×10^{-24}

One-year lag analysis	Excluding NAFLD events within the first year of follow-up	401,126	5,502	5,318,942.381	1.25 (1.20-1.31)	1.94×10^{-33}
Two-year lag analysis	Excluding NAFLD events within the first two years of follow-up	400,701	5,077	5,310,486.927	1.24 (1.18-1.30)	6.52×10^{-25}
Baseline-year exposure plus one-year lag	Baseline-year benzene exposure after excluding first-year NAFLD events	401,126	5,502	5,318,942.381	1.21 (1.16-1.27)	3.16×10^{-20}

Notes: Hazard ratios (HRs) and 95% confidence intervals (CIs) were estimated per IQR increment in benzene exposure using Cox proportional hazards models. The baseline-year exposure analysis used annual benzene concentration in the year of the first UK Biobank assessment as an alternative exposure metric. Lag analyses excluded participants who developed NAFLD within the first one or two years of follow-up. Models were adjusted for age, sex, ethnicity, BMI, education level, smoking status, alcohol drinking status, physical activity, household income, healthy diet score, residential distance to the coast, Townsend Deprivation Index, hypertension, diabetes, asthma, and sleep duration. NAFLD, nonalcoholic fatty liver disease; BMI, body mass index; CI, confidence interval; HR, hazard ratio; IQR, interquartile range.

Supplementary Table 23. Sensitivity analyses of mediation results addressing temporal ordering of benzene exposure, biological aging biomarkers, and incident NAFLD.

Sensitivity analysis	Participants / events	Mediator	Natural direct effect HR (95% CI)	Natural indirect effect HR (95% CI)	Proportion mediated, % (95% CI)	P for indirect effect
Primary mediation analysis	401,472 / 5,848	PA	1.1818 (1.1796-1.1849)	0.9985 (0.9959-1.0003)	-0.9 (-2.5, 0.2)	0.096
		HD	1.1739 (1.1692-1.1764)	1.0052 (1.0030-1.0093)	3.1 (1.8, 5.6)	<0.001
		AL	1.1751 (1.1726-1.1766)	1.0042 (1.0029-1.0063)	2.5 (1.7, 3.8)	<0.001
		ABA	1.1673 (1.1615-1.1708)	1.0109 (1.0079-1.0160)	6.5 (4.7, 9.6)	<0.001
Baseline-year benzene exposure	401,472 / 5,848	PA	1.1460 (1.1408-1.1517)	0.9991 (0.9968-1.0012)	-0.7 (-1.7, 0.6)	0.182
		HD	1.1402 (1.1347-1.1461)	1.0038 (1.0017-1.0064)	2.8 (1.1, 4.2)	0.009
		AL	1.1411 (1.1356-1.1468)	1.0034 (1.0018-1.0056)	2.5 (1.3, 3.6)	<0.001
		ABA	1.1348 (1.1286-1.1412)	1.0087 (1.0058-1.0128)	6.4 (3.8, 8.4)	<0.001
Excluding first-year NAFLD events	401,126 / 5,502	PA	1.1760 (1.1705-1.1814)	0.9988 (0.9964-1.0010)	-0.6 (-2.0, 0.5)	0.118
		HD	1.1688 (1.1632-1.1745)	1.0050 (1.0024-1.0084)	3.0 (1.5, 5.1)	0.002

		AL	1.1695 (1.1641-1.1752)	1.0041 (1.0025-1.0062)	2.4 (1.5, 3.7)	<0.001
		ABA	1.1618 (1.1556-1.1684)	1.0104 (1.0073-1.0151)	6.5 (4.4, 9.1)	<0.001
Excluding first two-year NAFLD events	400,701 / 5,077	PA	1.1672 (1.1612-1.1735)	0.9990 (0.9968-1.0011)	-0.7 (-1.6, 0.6)	0.164
		HD	1.1604 (1.1540-1.1668)	1.0048 (1.0021-1.0080)	3.1 (1.3, 4.9)	0.004
		AL	1.1610 (1.1547-1.1675)	1.0039 (1.0022-1.0060)	2.5 (1.4, 3.6)	<0.001
		ABA	1.1537 (1.1470-1.1608)	1.0098 (1.0068-1.0144)	6.4 (4.1, 8.8)	<0.001
Same-address ⩾10 years	292,614 / 4,245	PA	1.1805 (1.1732-1.1881)	0.9986 (0.9957-1.0012)	-0.8 (-2.6, 0.4)	0.137
		HD	1.1720 (1.1646-1.1797)	1.0055 (1.0027-1.0090)	3.3 (1.6, 5.5)	0.001
		AL	1.1736 (1.1660-1.1810)	1.0043 (1.0024-1.0067)	2.6 (1.6, 4.1)	<0.001
		ABA	1.1650 (1.1568-1.1734)	1.0112 (1.0078-1.0168)	6.7 (4.6, 9.9)	<0.001

Notes: Natural direct and indirect effects are presented on the hazard-ratio scale. Proportion mediated and 95% confidence intervals were estimated using 5,000 bootstrap resamples. The baseline-year exposure analysis used benzene concentration in the year of the first UK Biobank assessment as an alternative exposure metric. Lag analyses excluded participants who developed NAFLD within the first one or two years of follow-up. The same-address analysis was restricted to participants who had lived at the same residential address for at least 10 years. Models were adjusted for age, sex, ethnicity, BMI, education level, smoking status, alcohol drinking status, physical activity, household income, healthy diet score, residential

distance to the coast, Townsend Deprivation Index, hypertension, diabetes, asthma, and sleep duration. PA, phenotypic age; HD, homeostatic dysregulation; AL, allostatic load; ABA, accelerated biological aging; NAFLD, nonalcoholic fatty liver disease; CI, confidence interval; HR, hazard ratio.

Supplementary Table 24. Data split for the machine-learning analysis in the ICD-defined NAFLD cohort.

Dataset	Participants	NAFLD events	Event rate
Eligible ICD-defined cohort	401,472	5,848	1.46%
Training set	281,030	4,094	1.46%
Validation set	120,442	1,754	1.46%

Notes:The cohort was randomly split into training (70%) and validation (30%) sets with stratification by NAFLD event status. Hyperparameters were tuned using stratified 5-fold cross-validation within the training set. Class weights were used during model training to address class imbalance.

Supplementary Table 25. Validation discrimination metrics for 10 machine-learning models.

Model	ROC AUC (95% CI)	PR AUC	Accuracy	Sensitivity	Specificity	Balanced accuracy
GBM	0.753 (0.728, 0.778)	0.682	0.705	0.662	0.736	0.699
Logistic	0.748 (0.723, 0.773)	0.681	0.708	0.612	0.776	0.694

CatBoost	0.746 (0.722, 0.771)	0.68	0.693	0.722	0.672	0.697
NeuralNetwork	0.743 (0.718, 0.768)	0.688	0.679	0.756	0.624	0.69
Xgboost	0.739 (0.714, 0.764)	0.675	0.678	0.754	0.623	0.688
SVM	0.728 (0.703, 0.754)	0.655	0.667	0.681	0.657	0.669
LightGBM	0.724 (0.698, 0.750)	0.626	0.675	0.689	0.665	0.677
RandomForest	0.718 (0.692, 0.745)	0.662	0.686	0.581	0.761	0.671
Adaboost	0.702 (0.678, 0.726)	0.649	0.673	0.764	0.608	0.686
KNN	0.666 (0.639, 0.694)	0.6	0.62	0.617	0.623	0.62

Notes: Abbreviations: AUC, area under the curve; CI, confidence interval; GBM, gradient boosting machine; KNN, k-nearest neighbours; PR AUC, area under the precision–recall curve; ROC AUC, area under the receiver-operating-characteristic curve; SVM, support vector machine; XGBoost, extreme gradient boosting.

Supplementary Table 26. Threshold-specific classification metrics on the validation set.

Model	Threshold	Accuracy	Sensitivity	Specificity	Balanced accuracy	Precision	F1
GBM	0.469	0.705	0.662	0.736	0.699	0.643	0.653
Logistic	0.458	0.708	0.612	0.776	0.694	0.663	0.637

CatBoost	0.609	0.693	0.722	0.672	0.697	0.613	0.663
NeuralNetwork	0.374	0.679	0.756	0.624	0.69	0.591	0.663
Xgboost	0.499	0.678	0.754	0.623	0.688	0.59	0.662
SVM	0.449	0.667	0.681	0.657	0.669	0.588	0.631
LightGBM	0.39	0.675	0.689	0.665	0.677	0.597	0.64
RandomForest	0.485	0.686	0.581	0.761	0.671	0.636	0.607
Adaboost	0.237	0.673	0.764	0.608	0.686	0.583	0.662
KNN	0.411	0.62	0.617	0.623	0.62	0.541	0.576

Notes: For each model, the decision threshold that maximised Youden's index in the validation set was used to dichotomise the predicted probabilities. Accuracy, sensitivity, specificity, balanced accuracy, precision, and F1 score were computed at this model-specific threshold. Balanced accuracy is the mean of sensitivity and specificity; F1 is the harmonic mean of precision and sensitivity. All values were rounded to three decimal places. Abbreviations: F1, F1 score; GBM, gradient boosting machine; KNN, k-nearest neighbours; NAFLD, nonalcoholic fatty liver disease; SVM, support vector machine; XGBoost, extreme gradient boosting.

Supplementary Table 27. Sample selection flow for the urinary TTMA-HSI analytic sample

Selection step	Excluded since previous step	Remaining N
NHANES 2017-March 2020 pre-pandemic participants	NA	15560

Selection step	Excluded since previous step	Remaining N
Adults aged >=20 years	6328	9232
Eligible VOC biomarker data	5429	3803
After excluding pregnancy, definite hepatitis B/C, and excessive alcohol intake	460	3343
Positive urinary VOC2 subsample weight and non-missing urinary TTMA	1395	1948
HSI-defined hepatic steatosis outcome available	36	1912
Complete covariates for the fully adjusted TTMA-HSI model	231	1681

Supplementary Table 29. Adjusted odds ratios for HSI-defined hepatic steatosis by urinary TTMA

Pollution	Events	Total	Model a	Model b	P value
Urinary TTMA, ng/mL; per doubling	1,044	1,681	1.13 (1.04-1.23)	1.14 (1.04-1.24)	0.021
Q1 [6.94, 24.40]	246	422	ref	ref	
Q2 (24.60, 54.00]	271	419	1.40 (0.90-2.18)	1.34 (0.88-2.02)	
Q3 (54.30, 120.00]	258	420	1.27 (0.81-1.98)	1.19 (0.77-1.82)	

Pollution	Events	Total	Model a	Model b	P value
Q4 (121.00, 15800.00]	269	420	1.62 (1.07-2.47)	1.67 (1.09-2.55)	0.067

Notes: Model a adjusted for age and sex. Model b adjusted for age, sex, race/ethnicity, education level, family income-to-poverty ratio, smoking status, alcohol drinking status, and physical activity. TTMA denotes trans,trans-muconic acid, a urinary biomarker related to benzene exposure. TTMA was modeled per doubling after log2 transformation and by sample-specific quartiles.

Details of benzene assessment

Annual average concentrations of benzene (2006-2020) were collected from the Department for Environment, Food and Rural Affairs (DEFRA), a source known for gathering detailed near-surface air pollution data in the UK. The data from DEFRA are not only used in official government reports but also referenced in various academic publications [2-4]. Using a sophisticated air dispersion model, DEFRA produces annual concentration maps for a range of air pollutants at a high resolution of 1x1 km. This model incorporates data from the National Atmospheric Emissions Inventory and considers sources like dust resuspension and secondary inorganic aerosols. To validate the accuracy of the model, actual measurements from monitoring sites within DEFRA's Automatic Urban and Rural Network are integrated into the calibration process. DEFRA conducts thorough comparisons between modeled and measured air pollutant levels to ensure the reliability of the models, consistently finding strong agreement between the two datasets (https://uk-air.defra.gov.uk/assets/documents/reports/cat09/2402140956_2022_PCM_technical_report.pdf). For more detailed performance statistics of the model, interested parties can refer to https://uk-air.defra.gov.uk/data/pcm-data.

Contributions to ground level annual mean benzene concentrations from large point sources in the NAEI 2021 were estimated by modelling each

source explicitly using the atmospheric dispersion model ADMS 5.2 and sequential meteorological data for 2022 from the Weather Research and Forecasting Model (WRF). The WRF model is a next-generation numerical weather prediction modelling system developed by the US National Centre for Atmospheric Science (NCAR). This method accounts for spatial variation in meteorological parameters across the UK representing local dispersion characteristics within the PCM output. A total of 389 large point sources were modelled. Concentrations were calculated for a 99 km x 99 km square composed of a regularly spaced 1 km x 1 km resolution receptor grid. Each receptor grid was centred on the point source. For each large point source information was retrieved from the PCM stack parameters database. This database has been developed over a period of time under the Modelling of Ambient Air Quality (MAAQ) contract and its predecessors. The database is updated annually as required. Data sources for this database include a survey of Part A authorisation notices held by the Environment Agency.

Exposure accumulation window and temporal truncation rules: For each participant, the accumulation window for time-weighted average benzene exposure started in the calendar year of the baseline assessment and ended at the earliest of the following dates: (i) first diagnosis of NAFLD; (ii) death; (iii) loss to follow-up; (iv) the country-specific end of follow-up (October 31, 2022 for England; July 31, 2021 for Scotland; and February 28, 2018 for Wales); and (v) the last year for which annual benzene concentration data were available from DEFRA, namely 2020. For participants who remained under follow-up after 2020, the exposure accumulation window was truncated on December 31, 2020, because gridded benzene concentration estimates were not available beyond that year. This truncation rule ensured that exposure time was not extended beyond the occurrence of outcome or censoring events, thereby minimizing the potential for reverse causation.

For participants who moved residence during the accumulation window, the window was further divided according to residential address and calendar year. Each address-year unit was assigned the annual mean benzene concentration corresponding to the 1 km × 1 km grid cell of that address in that year. Individual time-weighted average benzene exposure was then calculated by weighting the benzene concentration of each address-year unit by the duration of residence within the accumulation window. Because the blood biomarkers used in the mediation analysis were measured at baseline, we conducted additional sensitivity analyses to address potential temporal misalignment between baseline biomarkers and subsequent exposure. Specifically, in the mediation sensitivity analysis, benzene exposure was replaced by the annual mean benzene concentration in the baseline assessment year (Supplementary Table 23). We also performed lag analyses by excluding participants who developed NAFLD within the first 1 year or 2 years of follow-up (Supplementary Table 22).

Mediation analysis details:

We performed mediation analyses to quantify the proportion of the association between long-term benzene exposure and NAFLD that was mediated by four biological aging indicators: PhenoAge, Homeostatic Dysregulation (HD), Allostatic Load (AL), and Accelerated Biological Aging (ABA). The estimation was based on the natural-effects framework proposed by Robins and Greenland (1992) and further formalized by VanderWeele (2015), which decomposes the total effect of an exposure on an outcome into the natural indirect effect (NIE), representing the component

transmitted through the mediator, and the natural direct effect (NDE), representing the component not transmitted through the mediator. All analyses were conducted in R software (version 4.2.1) using the mediation package. The proportion mediated and its 95% confidence interval were estimated using 5,000 bootstrap resamples, and all effect estimates were reported on the hazard-ratio (HR) scale.

The causal identification of the NDE and NIE relies on the following assumptions: (i) no unmeasured exposure-outcome confounding, meaning that benzene exposure is independent of the potential NAFLD outcomes conditional on measured covariates; (ii) no unmeasured mediator-outcome confounding, meaning that biological aging indicators are independent of the potential NAFLD outcomes conditional on benzene exposure and measured covariates; (iii) no unmeasured exposure-mediator confounding, meaning that benzene exposure is independent of the potential values of the biological aging indicators conditional on measured covariates; and (iv) no exposure-induced mediator-outcome confounding, meaning that there is no covariate affected by benzene exposure that also confounds the mediator-outcome relationship, corresponding to the cross-world independence assumption.

To approximate assumptions (i)-(iii), both the mediator and outcome models were adjusted for the same covariate set as Model B in the main analysis, including age, sex, ethnicity, body mass index, educational attainment, household income, smoking status, alcohol drinking status, physical activity, healthy diet score, residential distance to the coastline, Townsend Deprivation Index, hypertension, diabetes, asthma, and sleep duration. To address assumption (iv), we deliberately excluded variables that may lie downstream of the pathway “benzene exposure → biological aging → NAFLD”, particularly the serum biomarkers used to construct the biological aging indicators, to avoid overadjustment of the mediating pathway.

Because these assumptions cannot be fully verified in observational data, we conducted three sets of sensitivity analyses, as shown in

Supplementary Table 23. First, we replaced the time-weighted average benzene exposure with the annual mean benzene concentration in the baseline assessment year, ensuring that the exposure metric was temporally aligned with, and not later than, the baseline biomarker measurements. Second, we performed 1-year and 2-year lag analyses by excluding participants who developed NAFLD within the first 1 or 2 years of follow-up, respectively, to reduce the possibility of reverse causation. Third, we restricted the analysis to participants who had lived at the same residential address for at least 10 years before baseline, to minimize exposure misclassification due to residential relocation. The estimated mediation proportions from these sensitivity analyses were consistent with the main mediation analysis in both direction and magnitude, suggesting that the mediation findings were robust to reasonable deviations from the identification assumptions.

1 Miao, Z. *et al.* Identification of 90 NAFLD GWAS loci and establishment of NAFLD PRS and causal role of NAFLD in coronary artery disease. *HGG Adv* **3**, 100056 (2022). https://doi.org/10.1016/j.xhgg.2021.100056

2 Hill, W. *et al.* Lung adenocarcinoma promotion by air pollutants. *Nature* **616**, 159–167 (2023). https://doi.org/10.1038/s41586-023-05874-3

3 Clemens, T., Turner, S. & Dibben, C. Maternal exposure to ambient air pollution and fetal growth in North-East Scotland: A population-based study using routine ultrasound scans. *Environment international* **107**, 216–226 (2017). https://doi.org/10.1016/j.envint.2017.07.018

4 Tonne, C. & Wilkinson, P. Long-term exposure to air pollution is associated with survival following acute coronary syndrome. *European heart journal* **34**, 1306–1311 (2013). https://doi.org/10.1093/eurheartj/ehs480
